# Rational Strategy for Designing Peptidomimetic Small Molecules Based on Cyclic Peptides Targeting Protein–Protein Interaction between CTLA-4 and B7-1

**DOI:** 10.3390/ph15121506

**Published:** 2022-12-02

**Authors:** Kumiko Tsuihiji, Eiji Honda, Kanehisa Kojoh, Shizue Katoh, Tomonori Taguri, Atsushi Yoshimori, Hajime Takashima

**Affiliations:** 1 Drug Discovery Division, GeneFrontier Corporation, SHARP Kashiwa Building, 4F 273-1 Kashiwa, Kashiwa-shi 277-0005, Chiba, Japan; 2 PRISM BioLab Co., Ltd., C21F-4110, 26-1 Muraoka-Higashi 2-Chome, Fujisawa 251-8555, Kanagawa, Japan; 3 Chemoinformatics & AI Research Group, Institute for Theoretical Medicine, Inc., BW3M-20B, 26-1 Muraoka-Higashi 2-Chome, Fujisawa 251-0012, Kanagawa, Japan

**Keywords:** CTLA-4 B7-1 inhibitors, cyclic peptides, molecular docking, PepMetics, peptidomimetics, protein–protein interaction, PURE system, ribosome display, sequence mimic

## Abstract

Currently, various pharmaceutical modalities are being developed rapidly. Targeting protein–protein interactions (PPIs) is an important objective in such development. Cyclic peptides, because they have good specificity and activity, have been attracting much attention as an alternative to antibody drugs. However, cyclic peptides involve some difficulties, such as oral availability and cell permeability. Therefore, while small-molecule drugs still present many benefits, the screening of functional small-molecule compounds targeting PPIs requires a great deal of time and effort, including structural analysis of targets and hits. In this study, we investigated a rational two-step strategy to design small-molecule compounds targeting PPIs. First, we obtained inhibitory cyclic peptides that bind to cytotoxic T-lymphocyte-associated protein 4 (CTLA-4) by ribosomal display using PURE*frex*^®^ (PURE*frex*^®^*RD*) to get structure–activity relation (SAR) information. Based on that information, we converted cyclic peptides to small molecules using PepMetics^®^ scaffolds that can mimic the α-helix or β-turn of the peptide. Finally, we succeeded in generating small-molecule compounds with good IC_50_ (single-digit μM values) against CTLA-4. This strategy is expected to be a useful approach for small-molecule design targeting PPIs, even without having structural information such as that associated with X-ray crystal structures.

## 1. Introduction

Protein–protein interactions (PPIs) regulate various cellular processes, some of which are responsible for severe diseases such as cancer. Therefore, disease-related PPIs have recently attracted attention as drug-discovery targets. As pharmaceutical modalities have become increasingly diversified, many of their characteristics have been clarified. Small-molecule drugs are suitable for targets with deep, narrow, and rigid binding surfaces, such as receptors and enzymes, but they are generally inadequate for targets with flat, wide, and flexible binding surfaces, as is often the case in PPIs. By contrast, peptide drugs are currently being actively developed, along with in vitro screening technologies, as the next generation of drugs able to inhibit PPIs while maintaining the same level of specificity as antibodies despite their low molecular weight (0.5–5 kDa). Unfortunately, even though peptide drugs can be synthesized chemically, the manufacturing cost is higher compared to small-molecule drugs. Moreover, they are not easily administered orally and have difficulty penetrating into cells. The development of small-molecule drugs targeting PPIs remains an important challenge. The current mainstream method of small-molecule drug discovery targeting PPIs demands first that the structural information of target proteins complexed with small molecules be identified using various structural analysis methods, such as cryogenic electron microscopy, X-ray crystallography, and NMR analysis. None of these is an easy task [1,2,3]. Therefore, we attempted to establish a method that requires no structural analysis of the complexes to develop small-molecule drugs that use cyclic peptides against the target.

We chose cytotoxic T-lymphocyte-associated protein 4 (CTLA-4) as a target protein. CTLA-4 is expressed on the surface of T cells, activated by the antigen presentation from dendritic cells, and binds the related ligands B7-1 and B7-2 with higher affinity than T cell activator CD28, thereby suppressing T cell activation [4,5,6]. In other words, the blocked interaction of CTLA-4 with B7-1 and B7-2 makes it possible to enhance and sustain T cell activity [7]. In an earlier study, Fesik et al. used their fragment-based drug discovery (FBDD) method to obtain small molecules that bound to CTLA-4, but the method was found to be unsuitable for targets such as CTLA-4, which interacts on a wide surface, because it is a point-based approach for hot spots of the target [8].

As described herein, we established a rational two-step strategy for designing small molecules that integrates in vitro screening technology and peptidomimetics technology. First, we used PURE*frex*^®^RD [9], which combines a cell-free translation system and ribosome display, to obtain sequence information of cyclic peptides that inhibit the interaction between CTLA-4 and B7-1 together with structure–activity relation (SAR) information based on those peptides. Then, we identified small molecules that have inhibitory activity by screening a PepMetics^®^ scaffold mimicking the cyclic peptide sequence. The PepMetics^®^ scaffold precisely mimics the side chain C_α_-C_β_ bond of amino acids in α-helix or β-turn structures, thereby allowing the accurate transfer of the cyclic peptide sequence to small molecules. Furthermore, by implementing both molecular docking and structure-based drug design (SBDD) for small molecules selected by screening, we succeeded in creating a small-molecule compound with stronger inhibitory activity than the original cyclic peptide. The combination of PURE*frex*^®^RD and PepMetics^®^ scaffold presents benefits for the development of small-molecule drug discovery targeting PPIs without actual information from structural analysis of the target and binders.

## 2. Results

### 2.1. Selection and Affinity Maturation of Cyclic Peptide Ligands against CTLA-4

**Figure 1:**
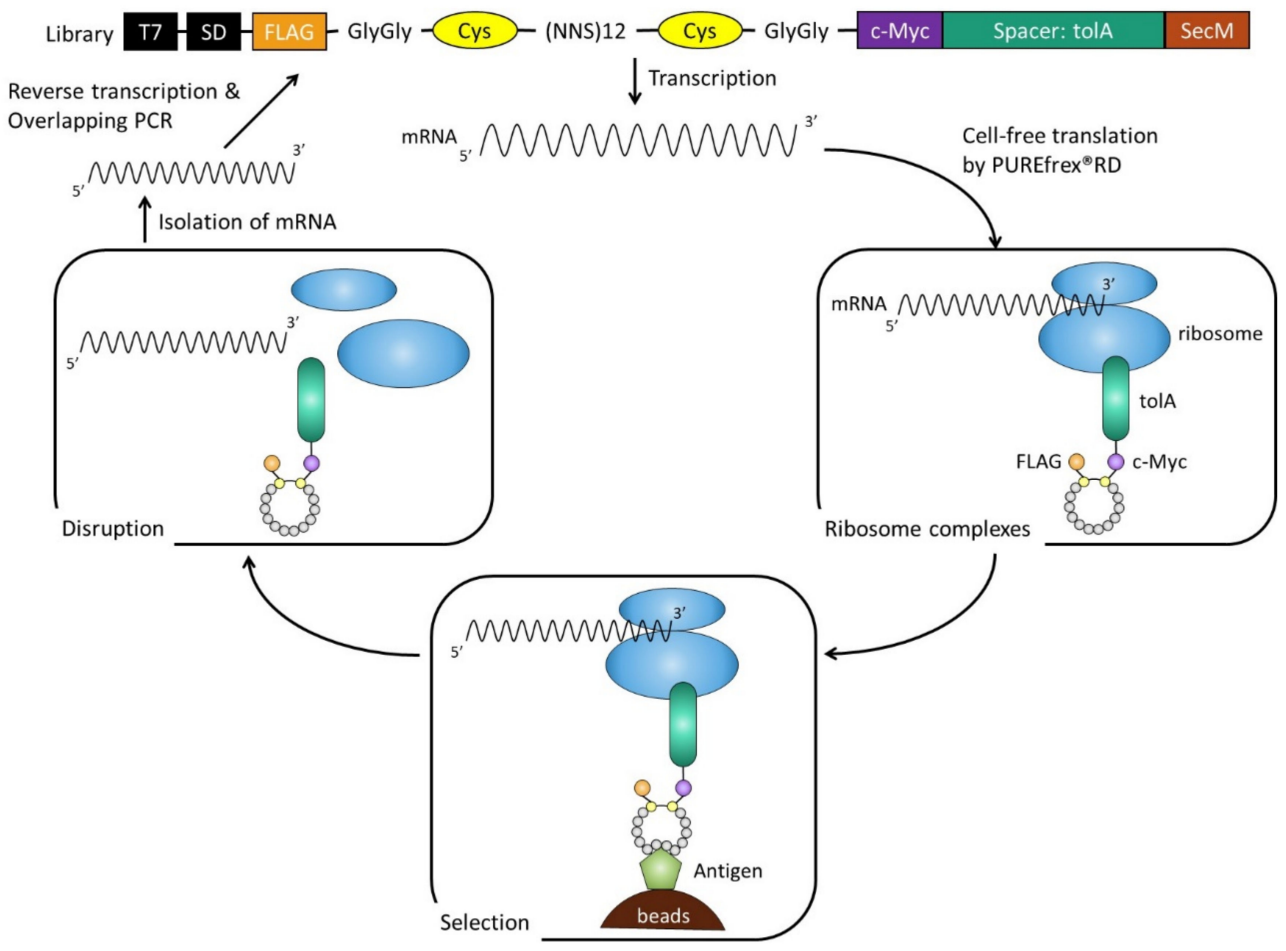
Ribosome display cycle. DNA encoding in library was first transcribed, and resulting mRNA was translated by PURE*frex*^®^RD cell-free translation system optimized for ribosome display, to give rise to mRNA–ribosome–peptide complexes through SecM arrest sequence, which acts within the ribosomal exit tunnel to stop translation. mRNA–ribosome–peptide complexes were used directly for selection against an immobilized target on beads. After selection, retained complexes were disrupted by the addition of EDTA. mRNA was isolated, reverse transcribed, and amplified for the next round.

**Figure 2:**
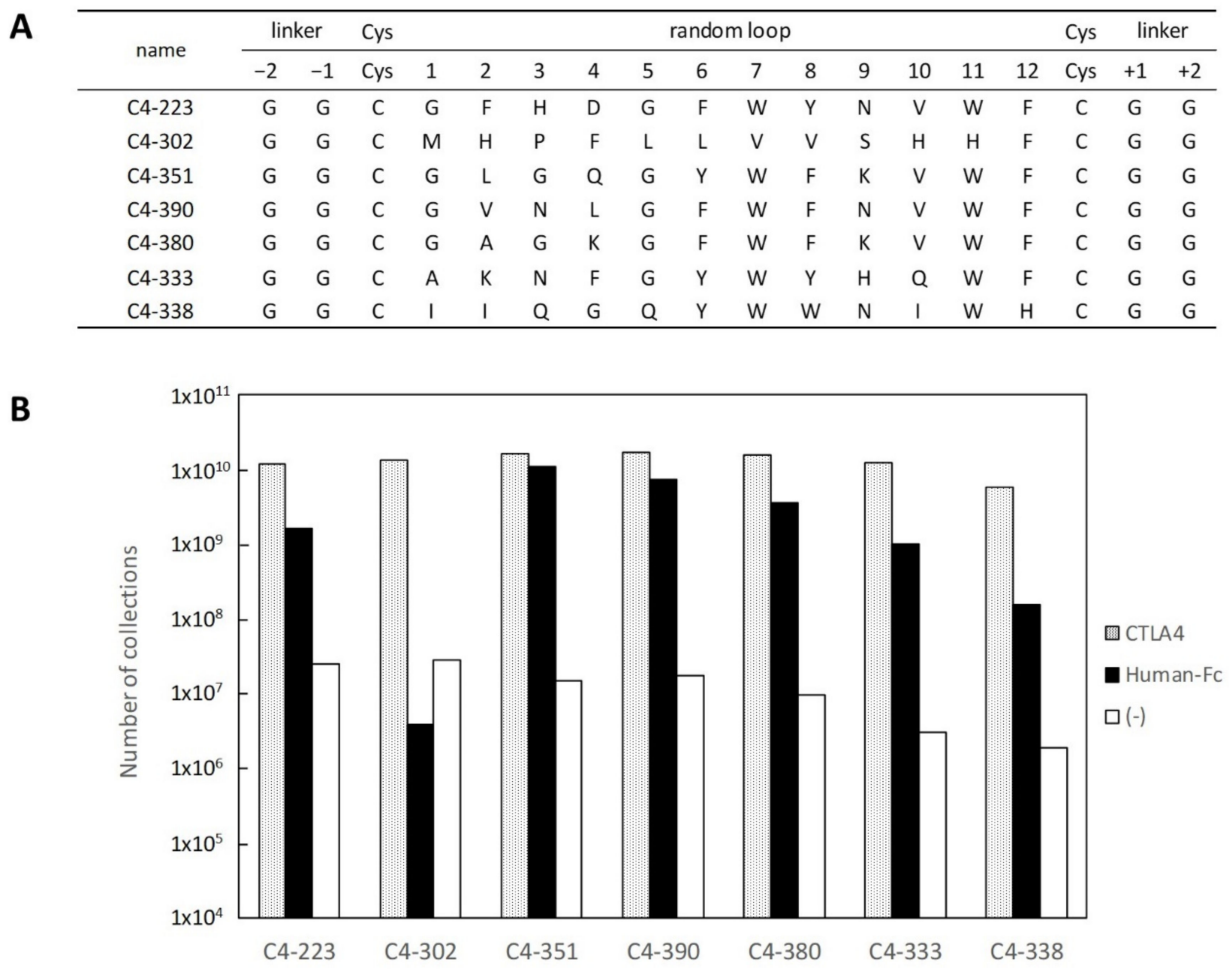
Sequences of selected clones and their binding specificity. (**A**) Selected amino acid sequences of clones from PURE*frex*^®^RD for CTLA-4-Fc. (**B**) Binding specificity of clones in (**A**) was evaluated as follows: mRNA–ribosome–peptide complex of each clone was reacted with each biotinylated protein (CTLA-4-Fc, dotted box; Human-Fc, black box; (-), white box) immobilized on streptavidin magnetic beads. Number of isolated mRNAs was determined by qPCR.

PURE*frex*^®^ is a reconstituted cell-free protein synthesis kit based on the PURE system [9]. This system consists of rigorously purified components related to transcription and translation, so it is possible to adjust its composition as needed. To screen cyclic peptides, we first optimized the reaction composition of PURE*frex*^®^RD for disulfide bond formation between cysteine residues, specifically by adding GSSG/GSH as redox reagents and DsbC as a chaperone. The library of cyclic peptides comprised 12 random amino acids flanked by two cysteines, a FLAG tag at the N-terminus, and a c-Myc-tolA-SecM sequence at the C-terminus for display on the ribosome (Figure 1). Quite often, TolA spacer is used as the unstructured domain in ribosome display, which allows cyclic peptides to be displayed on ribosomes [10]. The SecM stalling sequence arrests polypeptide chain elongation and generates stable mRNA–ribosome–peptide complexes. After three rounds of selection, we observed a dramatic increase in the number of isolated mRNA, as shown in Figure 1. Then, DNA that encodes peptides was PCR-amplified from the selected mRNA in the third round. In subsequent sequencing, seven sequences were identified (Figure 2A), and those with binding specificity to CTLA-4 were evaluated. The mRNA–ribosome–peptide ternary complex of each clone was reacted with biotinylated CTLA-4-Fc used in selection, biotinylated human IgG1-Fc with the same subclass as CTLA-4-Fc, and streptavidin beads. Figure 2B shows that six clones aside from C4-302 obtained mRNA of the eighth power or more not only for CTLA-4-Fc, but also for human IgG1-Fc. These results indicate that the six clones (aside from C4-302) specifically bound the Fc region of human IgG1. Moreover, no clone was found to bind to streptavidin beads. Only C4-302 was capable of binding specifically to CTLA-4.

**Figure 3:**
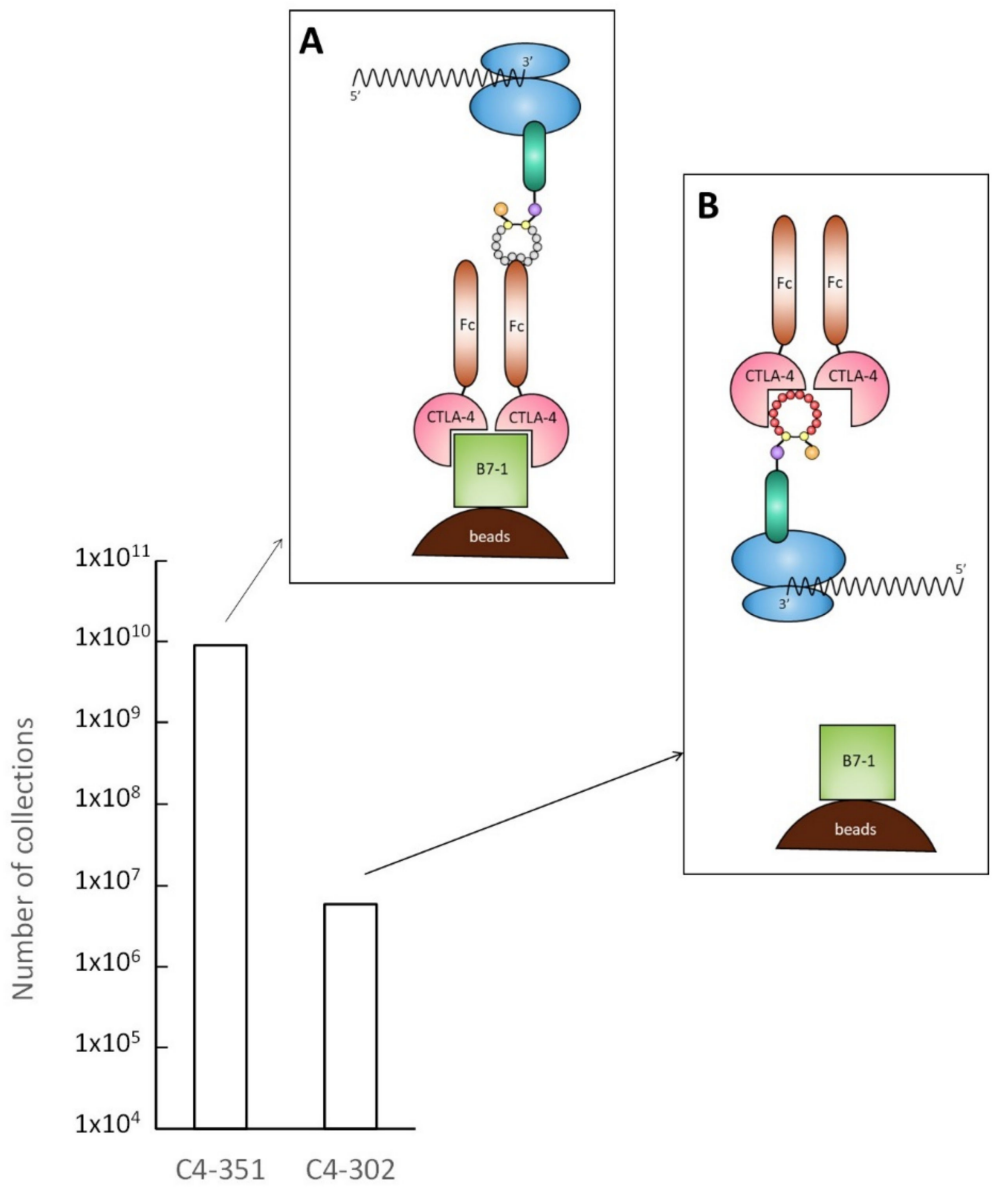
Inhibition of interaction between CTLA-4 and B7-1 by C4-302. Inhibitory activity of C4-302 was examined. C4-351, which was found to bind to Fc region of CTLA-4, was used as negative control. After mixing mRNA–ribosome–peptide complex of each clone with CTLA-4, B7-1 immobilized on streptavidin magnetic beads via biotin was added to the mixture. (**A**) Because C4-351 bound to the Fc region of CTLA-4, mRNA was collected at nearly 10^10^. (**B**) In C4-302, mRNA collection was reduced considerably to just under 10^7^. This finding suggests that the C4-302 mRNA–ribosome–peptide complex inhibited the interaction of CTLA-4 with B7-1.

**Figure 4:**
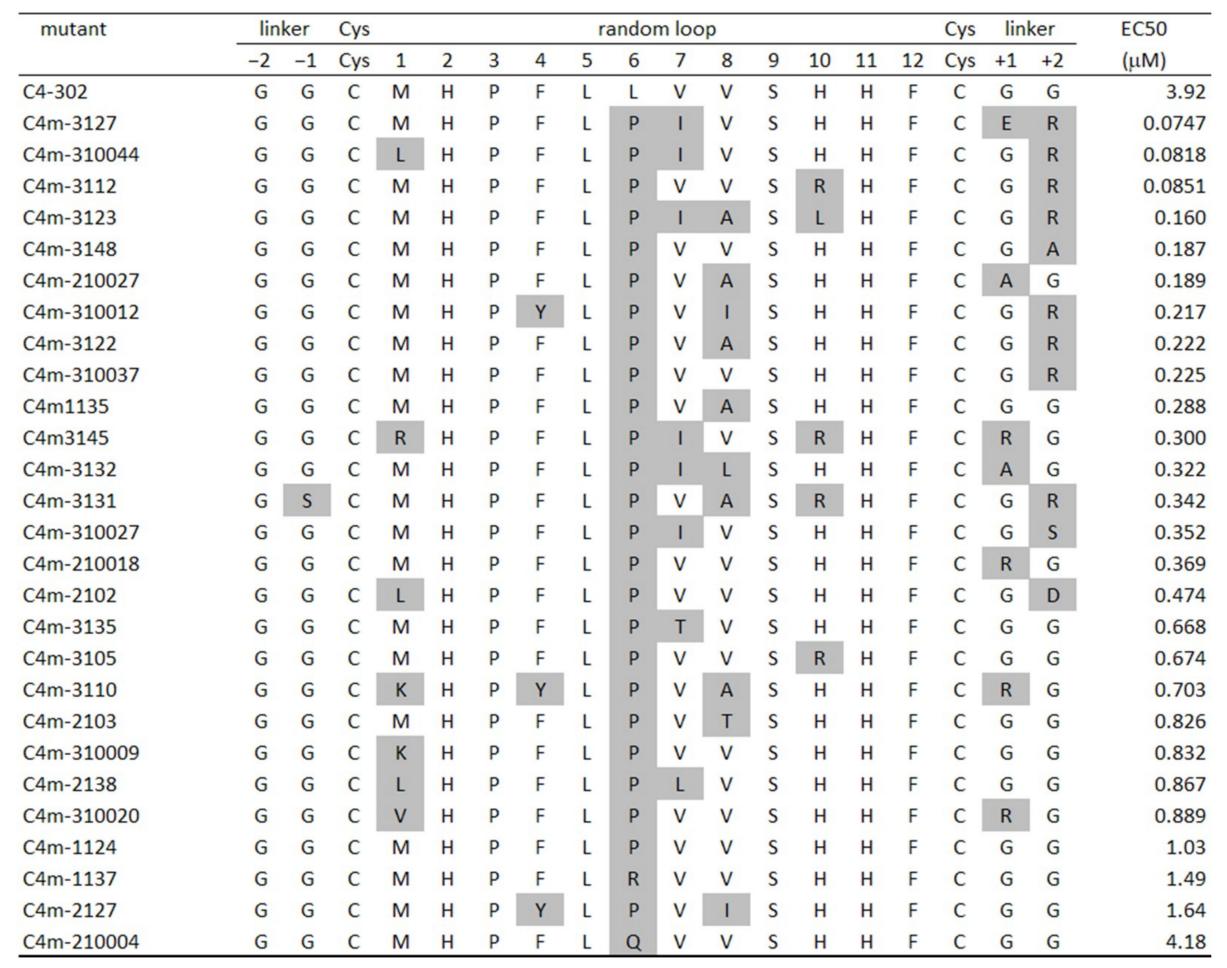
Affinity ranking of MBP fusion peptide using ELISA-based EC_50_ values. Amino acid sequences of many clones obtained from affinity maturation for C4-302 are shown. Residues highlighted in gray indicate mutated sites. Leu6 in random loop region of C4-302 was predisposed to mutate into proline. Although mutation in N-terminal GG linker was rarely observed, GG linker in C-terminus tended to mutate into hydrophilic residues such as arginine. ELISA-based EC_50_ values were determined using MBP fusion peptide that was overexpressed in *E. coli* as a fusion protein with an N-terminal MBP and then purified.

Using the method depicted in Figure 3, we tested whether C4-302 inhibits the interaction between CTLA-4 and B7-1. C4-351 was chosen as a negative control. The findings indicate that C4-351 bound to the Fc region of the CTLA-4 fusion protein. For that reason, it could not inhibit the CTLA-4 and B7-1 interaction. As a result, nearly 10^10^ of mRNA of C4-351 was recovered (Figure 3A). However, C4-302 greatly decreased mRNA recovery by a factor of 1000 compared to C4-351, suggesting that C4-302 binds to the binding surface between CTLA-4 and B7-1 and inhibits that interaction (Figure 3B). To assess its binding affinity toward CTLA-4, DNA encoding C4-302 was reconstituted for *E. coli* expression as a fusion protein, with MBP at the N-terminus and His-tag at the C-terminus, and purified. The half-maximal effective concentration (EC_50_) value of C4-302 was 3.92 μM (Figure 4). Because this was very low affinity for our purposes, we performed affinity maturation for C4-302.

The library for affinity maturation was prepared using error-prone PCR on a limited region from the GG linker behind the FLAG tag to the GG linker in front of the c-Myc tag. The library was selected under increasingly stringent conditions, with the addition of excessive non-biotinylated CTLA-4 in the washing process. After three rounds of maturation, the third-round pool was reconstituted as an MBP–cyclic peptide–His-tag fusion protein. The sequence analysis indicated the occurrence of many mutations, especially in each region of Leu6–Val8 and Gly+1–Gly+2 (Figure 4). Leu6 showed a high tendency to mutate into proline residue, which may have exceptional conformational rigidity because of the distinctive cyclic structure of the side chain. The mutation to a proline residue can restrict the degree of freedom of the polypeptide main chain and increase the structural stability. For these reasons, proline is thought to be preferred.

After the representative 27 mutants were prepared in fusion protein format, their EC_50_ values were examined using ELISA to evaluate their affinity. The EC_50_ value of C4m-1124 with the sole mutation of L6P was just one-third that of the original clone C4-302. In other words, the mutation of Leu6 to proline was affected only modestly to improve binding affinity toward CTLA-4. The EC_50_ of the best binder, C4m-3127 was 0.0747 μM, representing 50 times greater affinity than C4-302. Next, the inhibitory activity of the MBP–C4m-3127 peptide–His-tag fusion protein was tested using a kit (CTLA-4:B7-1[Biotinylated] Inhibitor Screening Assay; BPS Bioscience Inc. San Diego, CA, USA). The results confirmed the inhibitory activity of the fusion protein, with an IC_50_ value of 1.36 μM (data not shown).

### 2.2. Characterization of Selected Cyclic Peptide

**Figure 5:**
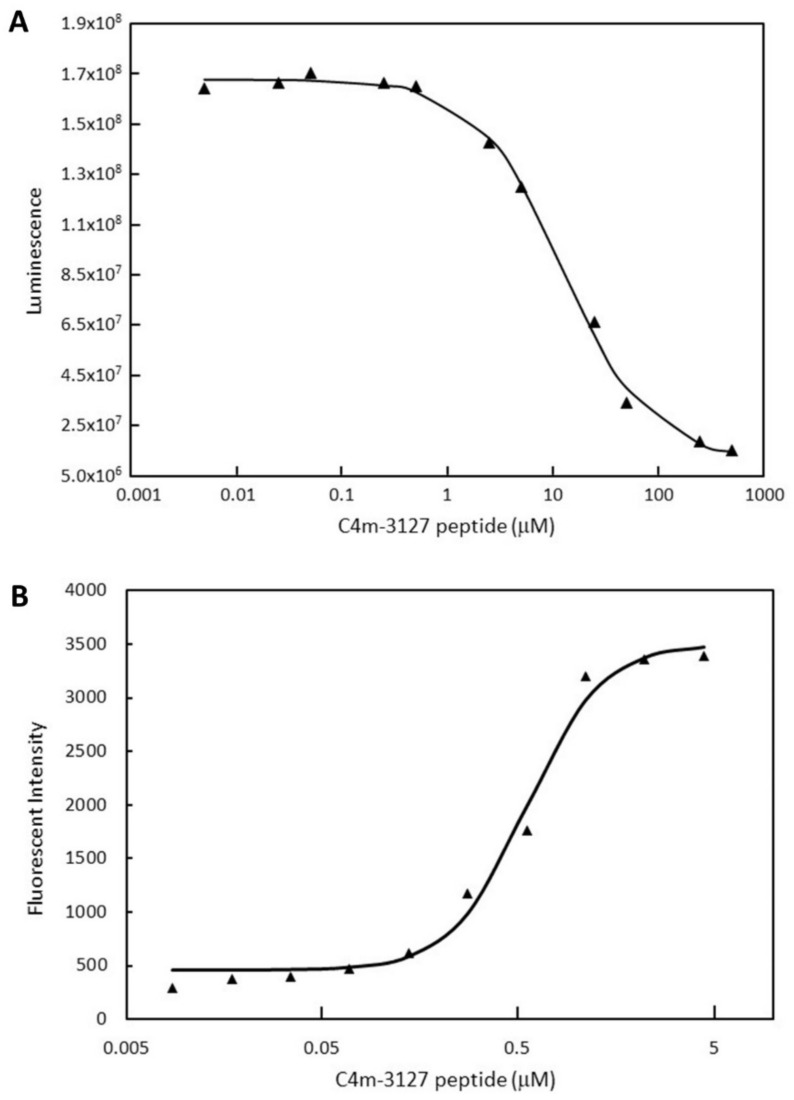
Characterization of C4m-3127 synthetic peptide. Triangles indicate luminescence (**A**) and fluorescence (**B**) values for each concentration of C4m-3127 synthetic peptide. (**A**) Half-maximal inhibitory concentration (IC_50_) value of synthetic cyclic peptide was measured using CTLA-4:B7-1[Biotinylated] Inhibitor Screening Assay Kit (BPS Bioscience Inc.) in 11-point two-fold serial dilution from 500 to 0.0050 μM by assay buffer. (**B**) Flow cytometry affinity analysis. CTLA-4 overexpressing CHO cell line was prepared at 1 × 10^5^ cells/100 mL and reacted with synthetic peptide C4m-3127 with biotin-PEG4-Lys to N-terminus. Peptide in 10-point two-fold serial dilution from 4.4 to 0.0086 μM by PBS was used for this measurement. Cells with biotinylated peptide bound to their surface were detected with streptavidin–IFluor 647 conjugate. Fluorescence intensity depending on the amount of bound peptide shown on vertical axis.

We prepared the C4m-3127 cyclic peptide using chemical synthesis and examined its properties. First, the inhibitory activity was tested using a kit (CTLA-4:B7-1[Biotinylated] Inhibitor Screening Assay, BPS Bioscience Inc.) (Figure 5A). Initially, we synthesized C4m-3127 peptide with Gly added to the N-terminus, but its solubility was so low it could not be measured. In a sequence of C4m-3127, the GG linker behind C-terminal Cys had mutated into Glu and Arg. The newly synthesized C4m-3127, with the highly hydrophilic amino acids Glu and Arg at the C-terminus, had improved solubility, which made measurement possible. A decrease in luminescence values with increasing peptide concentration was observed, indicating that synthesized peptide also inhibits the interaction between CTLA-4 and B7-1. Fitting the obtained inhibitory activity curve to the four-parameter logistic curve gave an IC_50_ value of 11.5 μM. The IC_50_ values for the C4m-3127 peptide were an order of magnitude higher than those for its fusion protein (1.36 μM). Although the hydrophilic amino acids were added to the C-terminus, the solubility of the synthesized C4m-3127 peptide still did not appear to be sufficient.

Subsequently, we used flow cytometry to investigate the binding activity of over-expressed CTLA-4 on the CHO cell surface (Figure 5B). Biotinylated C4m-3127 peptide with Glu and Arg added to the C-terminus was newly synthesized for detection with streptavidin–IFluor 647 conjugate. We obtained an EC_50_ value of 0.472 μM by measuring the change in fluorescence intensity with an increment of peptide concentration and fitting for the four-parameter logistic curve. The results revealed that the synthetic C4m-3127 peptide was able to bind to the expressed CTLA-4 on the cell surface.

### 2.3. Identification of Amino Acids Involved in Binding with CTLA-4

To obtain SAR information from the peptide, C4m-310044, with comparable affinity to C4m-3127, was chosen as the parental clone of alanine scanning mutagenesis (Figure 4). We generated 14 alanine mutants as an MBP–peptide–His-tag fusion protein (Supplementary Figure S1A), and systematically investigated the effects of alanine substitution by lysate ELISA (Supplementary Figure S1B). The six mutants (L1A, F4A, V8A, H10A, G(+1)A, and R(+2)A) showed the same level of absorbance with C4m-310044. The results show that these six residues were not involved in binding with CTLA-4. The residual eight mutants (H2A, P3A, L5A, P6A, I7A, S9A, H11A, and F12A) led to a marked decrease in absorbance. These eight residues were regarded as important for the interaction between C4m-310044 peptide and CTLA-4. Of the eight residues, six (H2, P3, L5, S9, H11, and F12) were highly conserved even after affinity maturation of C4-302 (Figure 4). These six amino acids were essential residues for binding with CTLA-4. The two other residues (P6 and I7) did not allow substitution for alanine, but did allow substitution for Leu, Pro, Arg, and Gln in the sixth position and to Val, Ile, Thr, and Leu in the seventh position. Neither of these two residues is involved in binding, but they seem to contribute to affinity.

The results indicate that 8 residues (H2, P3, L5, P6, I7, S9, H11, and F12) out of 12 positions in the loop region were important for both binding ability and affinity enhancement. We verified the amino acids allowed in the remaining four positions and the C-terminal GG linker, and whether the mutations in these positions improved affinity. A randomized library with NNS codon substitution for positions 1, 4, 8, and 10 in the loop region and two C-terminal amino acids was prepared. Then, two rounds of affinity maturation were conducted using PURE*frex*^®^RD. After the 137 selected clones were expressed as MBP–peptide–His-tag fusion proteins, their binding ability was evaluated using *E. coli* lysate ELISA (Supplementary Figure S2). The signal-to-noise ratio of the obtained clones was sufficiently high (>3) to maintain binding ability. Some of these clones were purified as MBP–peptide–His-tag fusion proteins, and their EC_50_ values were obtained. The lowest EC_50_ value was 0.0257 μM for the 5th4w-NN6-67 clone, which was about three times higher than that for C4m-3127. The top clones in terms of signal-to-noise ratio tended to appear for certain amino acids: Leu, Arg, and Trp in position 1; Phe, Trp, Tyr, and Val in position 4; Arg, Val, Trp, and Phe in position 8; and Val, Leu, and Trp in position 10. For the tolerated amino acid types in each position, the frequency of amino acids with bulky side chains tended to be high in all positions in the loop region (Supplementary Figure S3). The bulkiness of the side chains might have favored a better fit with the CTLA-4 binding surface. Position +1, immediately adjacent to the C-terminal cysteine, was replaced by either proline (41% of substitutions in position +1) or amino acids with a small side chain (Ala, Gly, and Ser, 39%). Proline is known to introduce bends or kinks into the peptide main chain. The presence of proline residue might facilitate the formation of a disulfide bond in the cyclic peptide. For amino acids with a small side chain, the smallness might be preferred so as to not interfere with the formation of disulfide bonds. As described above, we can get not only useful cyclic peptides, but also SAR information against the target with PURE*frex*^®^RD, which is expected to be useful for additional applications such as peptidomimetics.

### 2.4. Strategy for Transformation of Cyclic Peptides to Small Molecules

In general, it is known that a part of cyclic peptides form secondary structural elements, such as β-turn and α-helix, by transannular hydrogen bonds between main-chain amides (CO···NH) [11]. Secondary structural elements that fix the direction of their amino acid side chains are anticipated to be an important factor in the interaction between a cyclic peptide and its target protein. Many examples of such interactions are reported. In particular, β-turn was identified as a secondary structural element in many cyclic peptides [11]. In terms of geometry, cyclization of linear peptides increases the propensity of β-turn formation [12], because cyclization enables the formation of β-turn by a hydrogen bond between the main chain amide NH group and amide carbonyl groups of two amino acids located nearby (*i* and *i*+2). Somewhere in the cyclic peptide sequence, a β-turn structure will form to play an important role in interactions with target proteins. Therefore, the key to transforming cyclic peptides to small molecules is the precision with which small molecules with corresponding side chains mimic the β-turn. Nevertheless, it is difficult to identify the three consecutive amino acid sequences that form the β-turn in the cyclic peptide without their X-ray binding structures. In most cases, we can obtain potential cyclic peptides, but cannot obtain their X-ray structures. Exploring conformational spaces for cyclic peptides computationally remains a challenge despite many efforts [13]. A new strategy must be developed to transform the binding sites of cyclic peptides to small molecules without the need for structural information such as X-ray co-crystal structures or computational structural predictions.

**Figure 6:**
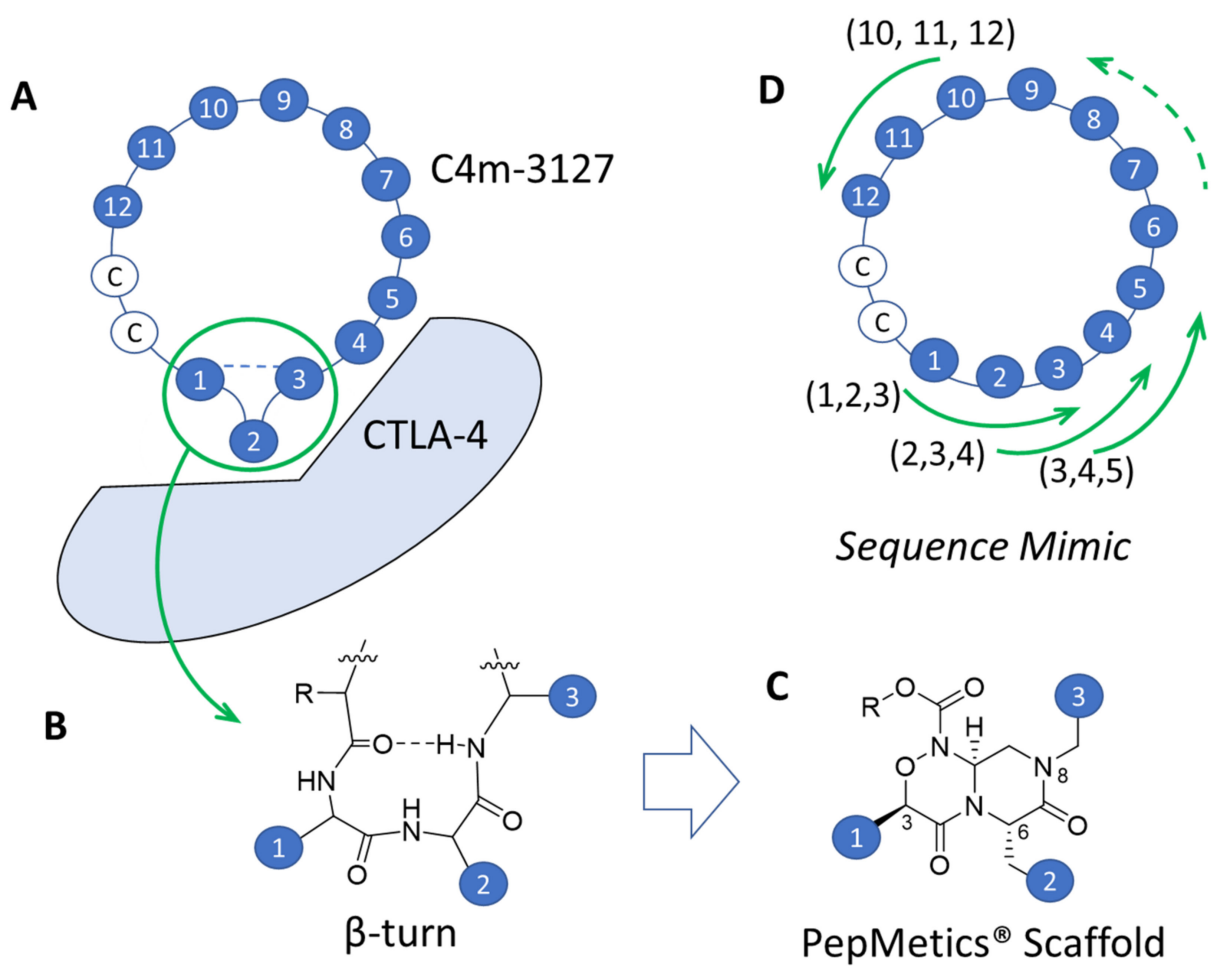
Sequence mimic: Strategy of transforming cyclic peptides to small molecules. (**A**) Conceptual diagram of interaction between C4m-3127 and CTLA-4. Dashed lines indicate hydrogen bonds. C, cysteine. Numbers from 1 to 12 correspond to sequence ^1^MHPFLPIVSHHF^12^. (**B**) Chemical structure of β-turn. Dashed lines indicate hydrogen bonds. (**C**) Representative PepMetics^®^ scaffold used in this research, among more than 40 kinds of scaffolds. For synthetic reasons, PepMetics^®^ scaffold cannot mimic histidine at the 3-position and proline at 6- and 8-positions. (**D**) Conceptual diagram of sequence mimic, with 10 patterns for mimicking sequences from (1, 2, 3) to (10, 11, 12). Because of the limitation of PepMetics^®^ for proline mimicking, this scaffold did not mimic the four sequences (M1, H2, P3), (H2, P3, F4), (F4, L5, P6), and (L5, P6, I7).

PepMetics^®^ is quite useful as a small-molecule scaffold to mimic β-turn and α-helix. Our earlier report explained its superiority to other peptide-mimicking scaffolds by peptide conformation distribution-plot (PCD-plot) analysis [14], and the findings of this study indicate that it is useful as a β-turn-mimicking scaffold (Figure 6B,C). If compounds that mimic all patterns of the three consecutive amino acid sequence of the cyclic peptide are synthesized sequentially from the end (Figure 6D), then one of them will bind to the site on the target protein where the cyclic peptide binds. Consequently, we can expect to identify the sequence that forms the β-turn of a cyclic peptide and mimic it with a small molecule without relying on structural information. We designated this strategy as “sequence mimic“.

The amino acid sequence selected for sequence mimic was C4m-3127 based on in vitro activity (Figure 4). C4m-3127 has two adjacent histidine at the 10- and 11-positions; H11 is important for in vitro potency, while H10 is replaceable with valine (Supplementary Figure S3). The amino acid sequence used for mimicking was the random loop of C4m-3127, for which histidine at the 10-position was changed to valine because of the synthetic limitation of the PepMetics^®^ scaffold (Figure 6C). The target sequence for sequence mimic was ^1^MHPFLPIVSVHF^12^.

**Figure 7:**
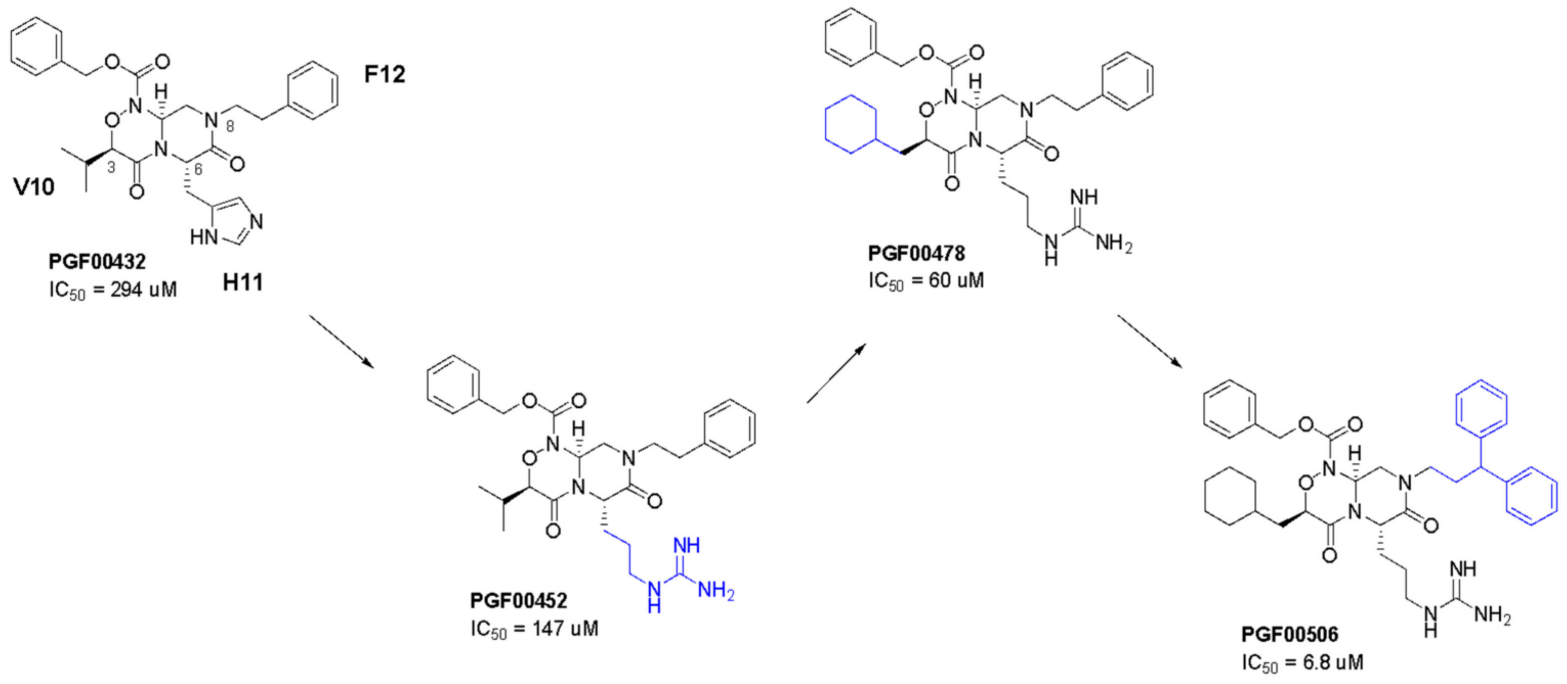
Chemical structures of key compounds. IC_50_ values show CTLA-4/B7-1 interaction inhibitory activity. Three amino acids (V10, H11, F12) on PGF00432 correspond to C4m-3127 H10V random loop: ^1^MHPFLPIVSVHF^12^.

Compounds with sequences that can be mimicked by PepMetics^®^ scaffold were synthesized. As evaluated by CTLA-4:B7-1[Biotinylated] inhibitor screening assay, PGF00432 (IC_50_ = 294 μM) was identified as a hit compound with weak potential. PGF00432 is a sequence-mimetic molecule of Val-His-Phe, with side chain structures of V10, H11, and F12 at the 3-, 6-, and 8-positions, respectively (Figure 7).

### 2.5. SBDD Approach

**Figure 8:**
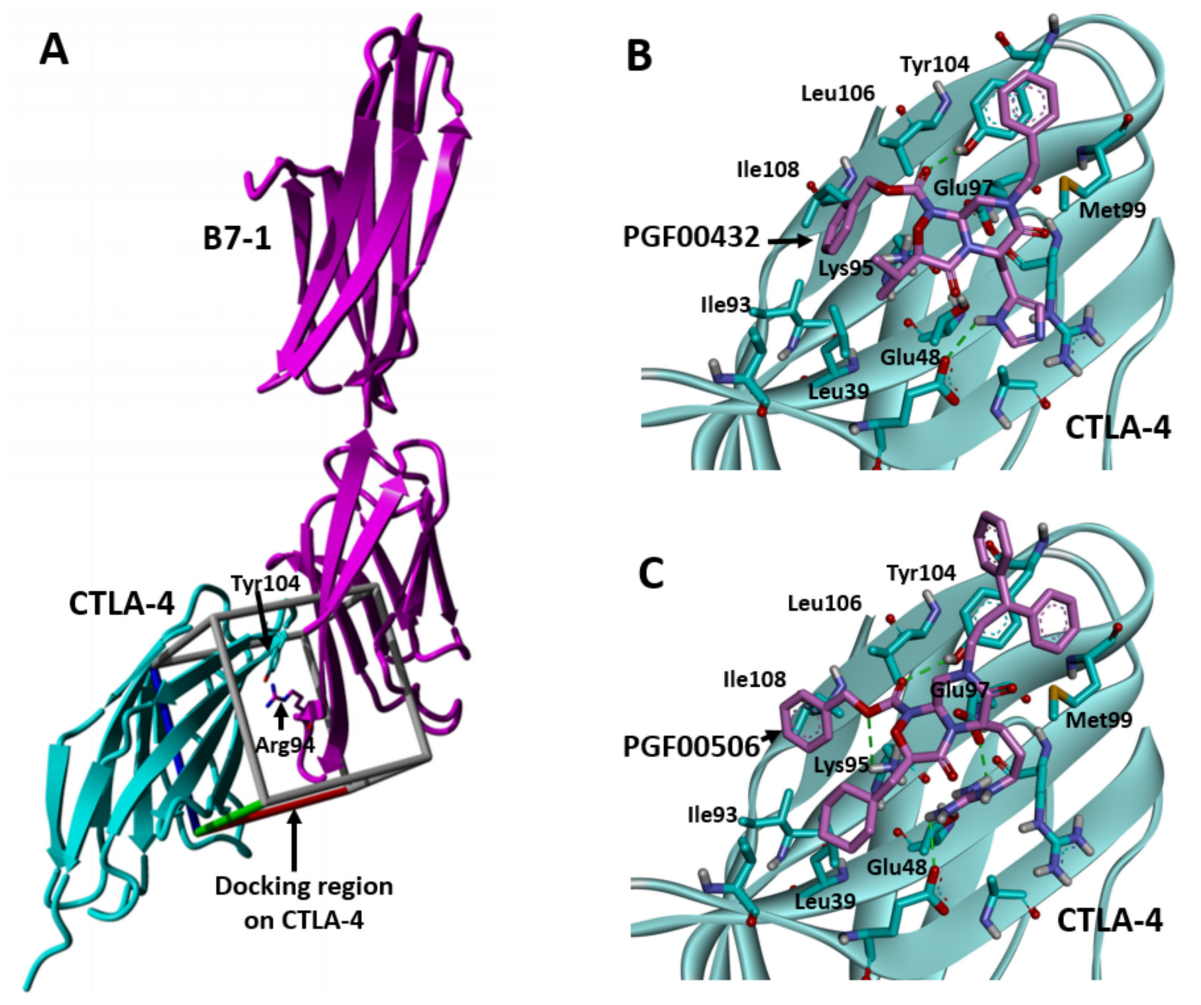
Predicted binding interaction of PGF00432 and PGF00506 with CTLA-4. (**A**) Crystal structure of B7-1/CTLA-4 complex (PDB code: 1I8L). Docking regions on CTLA-4 are derived from binding interface of the complex. (**B**) Predicted binding interaction of PGF00432 with CTLA-4. (**C**) Predicted binding interaction of PGF00506 with CTLA-4. Carbon, nitrogen, oxygen, and hydrogen are shown, respectively, in cyan (CTLA-4) or magenta (B7-1, PGF00432, and PGF00506), blue, red, and white. CTLA-4 and B7-1 are depicted as cyan and magenta ribbons, respectively. Dashed lines represent hydrogen bond interactions.

To increase inhibitory activity, docking simulation of PGF00432 with CTLA-4 was performed (Figure 8). Based on the results, which confirmed the inhibited interaction between CTLA-4 and B7-1 by C4-302 (Figure 3), this simulation was conducted in the search space including the surface at which CTLA-4 interacts with B7-1 (Figure 8A). The docking pose of PGF00432 with CTLA-4 indicated three points: (1) the histidine side chain of PGF00432 at the 6-position interacted with Glu48 of CTLA-4; (2) the valine side chain of PGF00432 at the 3-position partially filled the hydrophobic pocket formed by Ile93 and Leu39 of CTLA-4; and (3) the phenylalanine side chain of PGF00432 at the 8-position was located in the solvent region where CTLA-4 originally interacted with B7-1 (Figure 8B). According to these observations, we produced three strategies to increase in vitro activity: (1) search other basic side chain at the 6-position, (2) search the hydrophobic side chain at the 3-position to fill the hydrophobic pocket, and (3) search the bulky side chain at the 8-position to increase steric hindrance between CTLA-4 and B7-1.

As a result of derivative synthesis based on these strategies, the arginine side chain at the 6-position (PGF00462; IC_50_ = 147 μM), the cyclohexylmethyl group at the 3-position (PGF00478; IC_50_ = 80 μM), and the 3,3-diphenylpropyl group at the 8-position (PGF00506; IC_50_ = 6.8 μM) were found to be substructures that enhance in vitro activity (Figure 7). Furthermore, PGF00506 showed 43-fold inhibitory activity compared to PGF00432 and 1.7-fold inhibitory activity compared to C4m-3127 (IC_50_ = 11.5 μM) (Figure 5A). A docking simulation of PGF00506 with CTLA-4 indicated that the arginine side chain of PGF00506 at the 6-position interacted with Glu48 and Glu97 of CTLA-4 (Figure 8C). Because an interaction between the side chain at the 6-position and Glu97 was not observed in the case of PGF00432 (Figure 8B), it is expected that arginine is superior to histidine as a side chain at the 6-position. Histidine might be preferred in cyclic peptides because of its compatibility with surrounding amino acids, but it is speculated that downsizing to small molecules allows arginine, which has longer side chains, to bind even more strongly to CTLA-4. At the hydrophobic pocket formed by Ile93 and Leu39, the cyclohexylmethyl group of PGF00506 filled it better than the valine side chain of PGF00432. The substructure at the 8-position of PGF00506 was bulkier than that of PGF00432. This bulk might prevent the approach of B7-1 to CTLA-4.

### 2.6. Competitive ELISA

To ascertain whether PGF00506, the best compound found via the SBDD approach, binds to CTLA-4 on the same binding surface as the cyclic peptide C4m-3127, competitive ELISA was performed between PGF00506 and C4m-3127. Competition experiments were performed using biotinylated C4m-3127 peptide instead of biotinylated B7-1 in the CTLA-4:B7-1[Biotinylated] inhibitor screening assay. The biotinylated C4m-3127 peptide was optimized to a concentration of 30 μM in an earlier experiment. CTLA-4 immobilized on a 384-well plate was reacted with PGF00506 in 11-point two-fold serial dilution from 1000 to 0.98 μM by assay buffer. Then, the biotinylated peptides were added to wells for competitive reaction. After washing, the residual amounts of biotinylated peptides were detected using streptavidin-HRP. A decrease in luminescence values with increasing concentration of PGF00506 was observed and an IC_50_ value of 10.5 μM was confirmed (data not shown). These findings confirm that PGF00506 binds to CTLA4 on the same binding surface as the C4m-3127 peptide.

## 3. Discussion

As described herein, we introduced a rational two-step strategy to design small molecules using in vitro screening and peptidomimetics technology for targeting PPIs without the use of structural analysis such as X-ray crystallography. The first step is to find a cyclic peptide that interacts with a targeted protein, and to extract the sequence SAR information from the obtained cyclic peptide. In this way, we obtained cyclic peptides that bound to the extracellular domain of CTLA-4 and inhibited CTLA-4/B7-1 PPI by PURE*frex*^®^RD. PURE*frex*^®^RD is a much simpler in vitro display technology than phage/mRNA/cDNA display, and it can fully explore a highly diverse library easily and effectively. C4m-3127 was obtained as the representative cyclic peptide in this process. Peptides that inhibit the interaction between CTLA-4 and its ligand B7-1 have already been reported [15]. However, their inhibitory ability has only been reported qualitatively. It has been speculated that their affinity is weak. The peptides presented herein were cyclized using a disulfide bond, which increased their structural stability, resulting in an IC_50_ value of 11.5 μM. We also identified the sequence SAR information of the cyclic peptide’s random loop, including essential and non-essential amino acids and their positions for binding with CTLA-4, and acceptable amino acids instead of non-essential amino acids. Therefore, PURE*frex*^®^RD can be used to investigate the SAR between binder and target, which is useful for peptidomimetics in the second step of small molecule design.

In general, the SBDD and FBDD approaches to small molecules are suitable for PPI on “hot spots”, similar to a conventional “lock-and-key” model. However, they are unsuitable for PPI on a “surface”. For example, using the FBDD approach with a fragment library, Fesik’s group obtained no CTLA-4 binder [8] because the binding site between B7-1 and CTLA-4 is a wide “surface” compared to the small-molecule size. In contrast, the hit compound could be obtained using our approach because the SAR from the cyclic peptide library by PURE*frex*^®^RD included information of the interaction “surface” on CTLA-4. In addition, the PepMetics^®^ scaffold can reflect some information on the interaction “surface”. PURE*frex*^®^RD is suitable for this step because it can obtain not only hit compounds but also sequence SAR information by cyclic peptides.

The second step is to transform to small molecules from the cyclic peptide using the sequence mimic approach with the sequence SAR obtained in the first step. The obtained cyclic peptide C4m-3127 was transformed to small molecules using the sequence mimic approach. Compounds mimicking the three consecutive amino acids of C4m-3127 were synthesized considering the sequence SAR. PGF00432 was identified in this process as a hit compound with weak inhibitory activity (IC_50_ = 294 μM). Yoshida’s group reported on the transformation of cyclic peptide to small molecules. The salient aspect of their method was making pharmacophore based on the X-ray co-crystal structure of target protein and cyclic peptide [16]. By contrast, our approach requires no structural data of the complex of target protein or cyclic peptide, such as X-ray co-crystal structures. This obviation of data is a salient benefit of using sequence mimic. The sequence mimic concept enables the simple transfer of information from the cyclic peptide to the PepMetics^®^ scaffold.

In this case, the IC_50_ of the hit compound was a three-digit μM value, which was quite weak and near the detection limit of the in vitro assay. Therefore, it might have been missed by the screening of the small compound library from the beginning. Cyclic peptides with a length of 10–15 AAs have high sequential and structural diversity and good potential to generate useful hits against various targets. Therefore, starting from a cyclic peptide and using it as a guide for the design of a small compound leads to a “not to miss the hit” approach.

Finally, PGF00506 (IC_50_ = 6.8 μM), with 1.7-fold inhibitory activity of the original cyclic peptide C4m-3127, was discovered using the SBDD approach based on a docking simulation of PGF00432 with CTLA-4. The results of competition experiments indicated that the binding site of CTLA-4 and PGF00506 was identical to that of C4m-3127.

## 4. Materials and Methods

### 4.1. Construction of Cyclic Peptide Library

A cyclic peptide library for ribosome display was constructed with three oligonucleotides (synthesis by FASMAC, Kanagawa, Japan). Oligo1 included the essential T7 promoter, SD sequence as a ribosomal binding site, and FLAG-tag as an overlapping region with Oligo2. Oligo2 gave rise to a library sequence consisting of 12 randomized amino acids between two Cys residues. Overlapping regions with Oligo1 and Oligo3 were FLAG tag and c-Myc tag, respectively. Oligo3 coded for a c-Myc tag, TolA spacer, and SecM arrest sequence. Overlapping PCR for the construction of a full-length library was performed in two steps: (1) First, 1 pmol each of Oligo1, Oligo2, and Oligo3 was added to 500 μL of PCR reaction mixture without outer primer set and assembled as follows: 2 min at 94 °C, followed by 15 cycles of 10 s at 94 °C, 30 s at 58 °C, and 60 s at 68 °C. (2) Additional DNA polymerase and outer primer set were appended to the reaction mixture, amplified as follows: 2 min at 94 °C, followed by 10 cycles of 10 s at 94 °C, 30 s at 58 °C, and 60 s at 68 °C. The purified PCR product from agarose gel was transcribed to mRNA by the T7 Ribomax Express Large Scale RNA production system (Promega, Madison, WI, USA). The resulting mRNA was purified using NucleoSpin RNA clean-up (TaKaRa Bio, Shiga, Japan).

### 4.2. RD Selection and Affinity Maturation

In vitro translation was performed in a PURE*frex*^®^RD cell-free protein synthesis system optimized for S–S bond formation, to which was added GSSG/GSH as redox agents with the normal reaction mixture so that the final concentration was 3 mM each, and DsbC as chaperone so that its final concentration was 0.62 μM [17,18]. After 60 min at 37 °C, the translation reaction was stopped by dilution with ice-cold dilution buffer (50 mM Tris-HCl pH 7.5, 150 mM NaCl, 50 mM Mg(OAc)_2_, 0.5% Tween-20, 1 μg/mL *Saccharomyces cerevisiae* total RNA (Sigma Aldrich, St. Louis, MO, USA)). NanoLink streptavidin magnetic beads (Vector Laboratories, Inc., Newark, CA, USA), blocked using a blocking reagent, were washed with ice-cold washing buffer (50 mM Tris-HCl pH 7.5, 150 mM NaCl, 50 mM Mg(OAc)_2_, 0.5% Tween-20), and supplemented with 100 pmol of biotinylated human CTLA-4-Fc chimera (R&D Systems, Inc., Minneapolis, MN, USA) by EZ-Link™ NHS-PEO12-Biotin (Thermo Fisher Scientific, Waltham, MA, USA). After mixing the translation mixture and streptavidin beads with biotinylated CTLA-4 and rotating the mixture for 1 h at 4 °C, beads were isolated using a magnet. After 20 washes with ice-cold washing buffer, the retained ribosomal complexes were dissociated with elution buffer (50 mM Tris-HCl pH 7.4, 150 mM NaCl, 50 mM EDTA). The released mRNA was recovered using NucleoSpin RNA clean-up. Purified mRNA was quantified using qPCR (Light Cycler 480, Roche Diagnostics GmbH, Vienna, Austria). Then, reverse transcription PCR was performed using a kit (Transcriptor High Fidelity cDNA Synthesis Kit, Sigma Aldrich Co., St. Louis, MO, USA). After in vitro transcription of PCR products, the resultant mRNA library was used for the next round of ribosome display.

A library for affinity maturation was prepared using error-prone PCR [19] such that mutations occurred between the FLAG tag and c-Myc tag. The results show that mutations also occurred in the GG linker behind the FLAG tag sequence and the GG linker in front of the c-Myc tag. The error-prone library was constructed using a previously described overlapping PCR method. Error-prone PCR was implemented after each selection round. Affinity maturation was conducted with the following modification for RD selection: Biotinylated CTLA-4 was immobilized on streptavidin beads, altering the additive amount at each round: 10 pmol in the first round, 2 pmol in the second round, and 1 pmol in the third round. For off-rate selection, 1 mL washing buffer containing 1 mM non-biotinylated CTLA-4 was added to the washing process and was rotated for an appropriate time (2 h in the first round, 10 h in the second round, 67 h in the third round) at 4 °C. Subsequently, the beads were washed with ice-cold washing buffer, and mRNA was recovered using a method described earlier.

### 4.3. Cloning and Inhibitory Activity Evaluation

The mRNA pool after the third-round selection was subcloned into a pET-based vector to express the maltose-binding protein (MBP)–peptide fusion proteins and transformed into BL21(DE3) competent *E. coli* cells. Individual clones were picked and sequenced with an appropriate primer. Some enrichment clones in the third round were reconstructed for ribosome display format. The binding specificity for CTLA-4 was evaluated using the same method as that described above.

The inhibitory activity of the obtained clones was evaluated using the following method: First, the mRNA–ribosome–peptide ternary complex and non-biotinylated CTLA-4 were mixed for 1 h at 4 °C, then the mixture was reacted with streptavidin beads in conjunction with biotinylated B7-1 for 1 h at 4 °C. After 20 washes with ice-cold washing buffer, mRNA was recovered from the dissociated ternary complex, then subsequently quantitated using qPCR (Light Cycler 480, Roche Diagnostics GmbH, Vienna, Austria).

### 4.4. Overexpression and Purification of Cyclic Peptide–MBP Fusion

Peptides with binding specificity for CTLA-4 were overexpressed as fusion proteins with MBP at the N-terminus and His-tag at the C-terminus. After BL21(DE3) *E. coli* cells were transformed, the fusion constructs were grown in 200 mL of 2xYT/50 mg/mL ampicillin at 37 °C until an OD_600_ of 0.5–0.8 was reached. Then, IPTG (0.5 mM) was added. The broth was incubated at 25 °C with shaking overnight. After the cell broth was centrifuged, the pellet was resuspended in 20 mM Tris-HCl pH 7.5/500 mM NaCl and sonicated (Bioruptor UCD-250, Sonicbio Co. Ltd., Kanagawa, Japan). The sample was centrifuged, then the supernatant was purified on Ni Sepharose^TM^ 6FF (Cytiva, Tokyo, Japan). The purified sample was buffer exchanged to PBS by dialysis and quantified using a kit (BCA Protein Assay Kit, Thermo Fisher Scientific, Waltham, MA, USA).

### 4.5. ELISA-Based Determination of EC_50_ Value

CTLA-4 (5 μg/mL) was coated onto a NUNC^®^ Maxisoap^TM^ 384 well plate and incubated at 4 °C overnight. After the wells were washed twice with TBS/0.05% Tween-20, they were blocked with 100 μL of TBS/0.05% Tween-20/5% skim milk by incubating at RT for 1 h with shaking. The wells were then washed twice, as before. Purified MBP–peptide–His-tag fusion constructs were prepared in a 15-point two-fold serial dilution from 14 μM to 0.85 nM by PBS with 0.5% BSA. The diluted samples were added to CTLA-4-immobilized wells and incubated for 1 h with shaking, followed by washing five times and incubation with anti-FLAG M2^®^ peroxidase antibody (1:10,000; Sigma Aldrich Co., St. Louis, MO, USA) for 1 h. After the wells were washed five times, 1x TMB substrate solution was added, and they were incubated for a few minutes until a blue color developed. Then, 2 N hydrochloric acid was added to stop the reaction. The absorbance was measured at 450 nm/650 nm with a microplate reader (Infinite F200, Tecan Japan Co., Ltd., Kanagawa, Japan). The absorbance versus the concentration of MBP–peptide–His-tag fusion was found. The midpoint concentration was determined as the half-maximal effective concentration (EC_50_) value.

### 4.6. Affinity Measurement of Synthetic Cyclic Peptide

A cyclic peptide with a low EC_50_ value was synthesized by adding glycine to the N-terminus at Toray Research Center. The half-maximal inhibitory concentration (IC_50_) value of synthetic cyclic peptide was found using a kit (CTLA-4:B7-1[Biotinylated] Inhibitor Screening Assay, BPS Bioscience Inc., San Diego, CA, USA).

The binding activity of the cyclic peptide for overexpressed CTLA-4 on the CHO cell surface was evaluated using flow cytometry affinity analysis. Because the measurement requires a detection tag, cyclic peptide with biotin-PEG4-Lys added to the N-terminus was particularly synthesized. The CTLA-4 overexpressing CHO cell line was prepared in a 1 × 10^5^ cells/100 μL reaction and reacted with the biotinylated cyclic peptide in a 10-point two-fold serial dilution from 4.4 to 0.0086 μM using PBS. Detection was performed on streptavidin–IFluor 647 conjugate (GenScript Japan Corp., Tokyo, Japan.).

### 4.7. Chemistry

#### 4.7.1. General Methods

^1^H-and ^13^C-NMR spectra were recorded at operating frequencies of 300 and 75 MHz, respectively, using tetramethylsilane (TMS) as the internal resonance shift standard. All chemical shifts (δ) are reported in parts per million (ppm) and coupling constants (*J*) in Hertz. The spectral data were analyzed using MNOVA software. Mass spectra were obtained using a mass spectrometer (Shimadzu LCMS-2020 UFLC/MS System, Shimadzu Corp., Kyoto, Japan). Flash chromatography was performed on a Purif™ System (Shoko Scientific Co. Ltd., Kanagawa, Japan) using silica gel (30–50 μm particle size). Preparative HPLC was performed (column: C30-UG 25 mmID*150 mmL, 5 pm; mobile phase: acetonitrile and water with 0. 10% *v*/*v* acetic acid; FractionLynx System, Waters Corp., Milford, MA, USA). HRMS spectra were recorded using electrospray ionization time-of-flight measurement (ESI-TOF; maXis 4G, Bruker Analytik GmbH, Rheinstetten, Germany).

Compounds **2** and **7** were commercially available, and compounds **1** (CAS: 94508-09-5), **5** (CAS: 2767029-73-0), and **12** (CAS: 2758680-15-6) were known. They were synthesized by methods reported in WO2022075486. Solvents and other commercially available reagents were used as received, without further purification or modification.

#### 4.7.2. Abbreviations

**Scheme 1:**
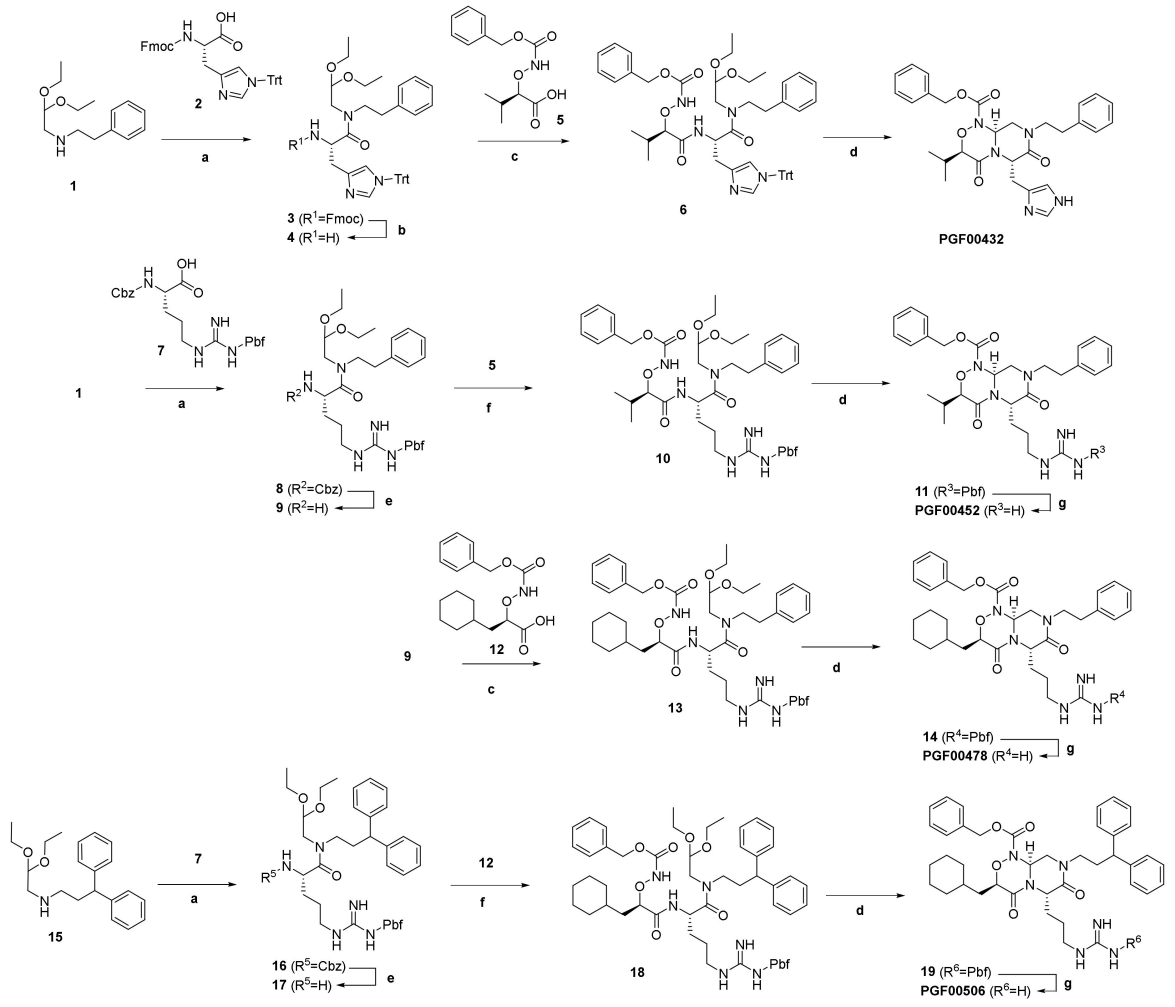
Synthesis of compounds PGF00432, PGF00452, PGF00476, and PGF00506. Reagents and conditions: (**a**) HATU, DIEA, THF, or DCM, 15 °C at RT for 14–18 h; (**b**) DBU, THF at RT for 17 h; (**c**) 4-methylmorpholine, DMT-MM, THF, or MeOH at RT for 20 h; (**d**) formic acid at RT for 20 h to 6 days; (**e**) Pd/C, H_2_, EtOH at RT for 72 h; (**f**) HATU, DIEA, DMF at RT for 0.5 h to 4 days; (**g**) TFA at RT for 21 h, or TFA–water (10:1) at RT to 60 °C for 2–3.5 h.

HATU: [dimethylamino(triazolo [4,5-b]pyridin-3-yloxy)methylene]-dimethyl-ammonium;hexafluorophosphate
DMT-MM: 4-(4,6-dimethoxy-1,3,5-triazin-2-yl)-4-methylmorpholin-4-ium chloride
DBU: 1,8-diazabicyclo [5.4.0]-7-undecene
DIEA: *N*,*N*-diisopropylethylamine
Trt: triphenylmethyl
Pbf: 2,2,4,6,7-pentamethyldihydrobenzofuran-5-sulfonyl
Fmoc: 9-fluorenylmethyloxycarbonyl
The synthetic routes and conditions are shown in Scheme 1.

#### 4.7.3. Synthesis of PGF00432

(*S*)-2-amino-N-(2,2-diethoxyethyl)-N-phenethyl-3-(1-trityl-1H-imidazol-4-yl)propanamide (**4**).

A mixture of 2,2-diethoxy-*N*-phenethyl-1-amine (**1**) (2.09 g, 8.8 mmol), *N*^α^-(((9*H*-fluoren-9-yl)methoxy)carbonyl)-*N*^τ^-trityl-L-histidine (**2**) (5.46 g, 8.8 mmol), HATU (6.70 g, 17.6 mmol), and DIEA (2.3 mL, 13.2 mmol) in THF (30 mL) was stirred at RT for 14 h. The mixture was added to water (20 mL), with extraction by EtOAc (20 mL). The organic phase was dried over anhydrous Na_2_SO_4_ and concentrated in vacuo. The residue was purified using column chromatography (SiO_2_, hexane/EtOAc = 9/1 to 0/1, gradient) to give crude (9*H*-fluoren-9-yl)methyl (*S*)-(1-((2,2-diethoxyethyl)(phenethyl)amino)-1-oxo-3-(1-trityl-1*H*-imidazol-4-yl)propan-2-yl)carbamate (**3**) (8.01 g) as a white amorphous powder. This material was dissolved in THF (30 mL), and added to DBU (3.32 mL, 22 mmol) at 0 °C. The reaction mixture was warmed gradually to RT, and was stirred at RT for 17 h. The reaction mixture was diluted with EtOAc (10 mL) and washed with diluted aq. HCl solution. The aqueous phase was extracted with EtOAc–MeOH (5:1, 5 × 10 mL). The combined organic phase was dried over anhydrous Na_2_SO_4_ and concentrated in vacuo. The residue was purified via column chromatography (SiO_2_, hexane/EtOAc = 9/1 to 0/1, gradient) to give **4** (4.4 g, 74% yield) as a brown oil.

LC/MS *m*/*z* 617.4 [M + H]^+^.

Benzyl (((*R*)-1-(((*S*)-1-((2,2-diethoxyethyl)(phenethyl)amino)-1-oxo-3-(1-trityl-1*H*-imidazol-4-yl)propan-2-yl)amino)-3-methyl-1-oxobutan-2-yl)oxy)carbamate (**6**).

A mixture of **4** (475 mg, 0.77 mmol), (*R*)-2-((((benzyloxy)carbonyl)amino)oxy)-3-methylbutanoic acid (**5**) (206 mg, 0.77 mmol), 4-methylmorpholine (0.17 mL, 1.54 mmol), and DMT-MM (320 mg, 1.16 mmol) in THF (8 mL) was stirred at RT for 20 h. To the mixture was added saturated aqueous NaHCO_3_ (5 mL), with extraction by EtOAc (10 mL). The organic phase was washed with brine (10 mL), dried over anhydrous Na_2_SO_4_, and concentrated in vacuo. The residue was purified using column chromatography (SiO_2_, hexane/EtOAc = 4/1 to 0/1, gradient) to give **6** (347 mg, 52% yield) as a colorless oil.

LC/MS *m*/*z* 867.3 [M + H]^+^.

Benzyl (3*R*,6*S*,9a*S*)-6-((1*H*-imidazol-4-yl)methyl)-3-isopropyl-4,7-dioxo-8-phenethylhexahydropyrazino [2,1-c][1,2,4]oxadiazine-1(6*H*)-carboxylate (PGF00432).

A mixture of **6** (347 mg, 0.40 mmol) and formic acid (1.5 mL) was left to stand at RT for 6 days. The mixture was concentrated in vacuo, and was azeotroped with CH_2_Cl_2_. The residue was purified using column chromatography (SiO_2_, EtOAc/MeOH = 1/0 to 17/3, gradient). The obtained material was purified by preparative HPLC (column: C30-UG-5, MeCN-H_2_O w/0.1% AcOH = 20 to 70%). After the collected fraction was concentrated in vacuo, the resulting aqueous solution was freeze-dried to give PGF00432 (188 mg, 88% yield) as a white powder.

^1^H NMR (300 MHz, CD_3_OD) δ 7.44–7.33 (m, 5H), 7.25–7.12 (m, 5H), 6.77 (s, 1H), 5.33–5.14 (m, 3H), 5.02–4.99 (m, 1H), 4.38 (s, 1H), 3.65–3.60 (m, 2H), 3.49 (t, *J* = 10.5 Hz, 1H), 3.14–3.12 (m, 1H), 2.99 (dd, *J* = 12.0 Hz, 3.0 Hz, 1H), 2.94–2.78 (m, 2H), 2.42–2.32 (m, 1H), 1.00 (d, *J* = 6.0 Hz, 3H), 0.62 (d, *J* = 6.0 Hz, 3H).

^13^C NMR (75 MHz, CDCl_3_) δ 166.11, 165.08, 153.70, 138.22, 135.20, 134.96, 128.80, 128.75, 128.72, 128.66, 128.42, 128.74, 84.90, 77.25, 68.89, 62.20, 55.40, 50.65, 49.64, 33.33, 28.74, 18.50, 15.87.

HRMS (EI) *m*/*z*: calcd. for C_29_H_33_N_5_O_5_ [M + H]^+^, 532.2560; found, 532.2537.

#### 4.7.4. Synthesis of PGF00452

(*S*)-2-amino-*N*-(2,2-diethoxyethyl)-5-(3-((2,2,4,6,7-pentamethyl-2,3-dihydrobenzofuran-5-yl)sulfonyl)guanidino)-*N*-phenethylpentanamide (**9**).

To a stirring mixture of **1** (35 g, 146 mmol), *N*^2^-((benzyloxy)carbonyl)-*N*^ω^-((2,2,4,6,7-pentamethyl-2,3-dihydrobenzofuran-5-yl)sulfonyl)-L-arginine (**7**) (82 g, 146 mmol), and DIEA (51 mL, 292 mmol) in DCM (400 mL) was added HATU(61 g, 161 mmol) in portions at 0 °C. The reaction mixture was stirred at 15 °C for 18 h, then was washed with water (3 × 200 mL), dried over anhydrous Na_2_SO_4_, filtered, and concentrated in vacuo. The residue was purified using column chromatography (SiO_2_, petroleum ether/ethyl acetate = 10/1 to 2/1) to give benzyl (*S*)-(1-((2,2-diethoxyethyl)(phenethyl)amino)-1-oxo-5-(3-((2,2,4,6,7-pentamethyl-2,3-dihydrobenzofuran-5-yl)sulfonyl)guanidino)pentan-2-yl)carbamate (**8**) (57 g, 50% yield) as a yellow oil. A part of this material (25 g, 33 mmol) was dissolved in EtOH (150 mL), to which was added Pd/C (3.0 g, 10% purity) under nitrogen atmosphere. The suspension was degassed under vacuum and purged with hydrogen several times. The mixture was stirred under hydrogen (15 psi) at 25 °C for 72 h. The reaction mixture was filtered, and the filtrate was concentrated under reduced pressure to give **9** (12 g, 57% yield) as a yellow solid.

LC/MS *m*/*z* 646.3 [M + H]^+^.

Benzyl (3*R*,6*S*,9a*S*)-3-isopropyl-4,7-dioxo-6-(3-(3-((2,2,4,6,7-pentamethyl-2,3-dihydrobenzofuran-5-yl)sulfonyl)guanidino)propyl)-8-phenethylhexahydropyrazino[2,1-c][1,2,4]oxadiazine-1(6*H*)-carboxylate (**11**).

A mixture of **9** (66 mg, 0.10 mmol), **5** (33 mg, 0.12 mmol), HATU (58 mg, 0.15 mmol), and DIEA (27 μL, 0.15 mmol) in DMF (1 mL) was stirred at RT for 4 days. To the mixture was added water (5 mL), with extraction by hexane-EtOAc (1:1, 5 mL). The organic phase was dried over anhydrous Na_2_SO_4_, and concentrated in vacuo to give benzyl (((*R*)-1-(((*S*)-1-((2,2-diethoxyethyl)(phenethyl)amino)-1-oxo-5-(3-((2,2,4,6,7-pentamethyl-2,3-dihydrobenzofuran-5-yl)sulfonyl)guanidino)pentan-2-yl)amino)-3-methyl-1-oxobutan-2-yl)oxy)carbamate (**10**) as a crude product. This material was dissolved in formic acid (1 mL) and left to stand at RT for 21 h. The mixture was concentrated in vacuo. The residue was diluted with EtOAc and washed with saturated aqueous NaHCO_3_. The organic phase was dried over anhydrous Na_2_SO_4_ and concentrated in vacuo to give **11** (66 mg, 80% yield) as a white powder.

LC/MS *m*/*z* 803.4 [M + H]^+^.

Benzyl (3*R*,6*S*,9a*S*)-6-(3-guanidinopropyl)-3-isopropyl-4,7-dioxo-8-phenethylhexahydropyrazino [2,1-c][1,2,4]oxadiazine-1(6*H*)-carboxylate (PGF00452).

A mixture of **11** (66 mg, 82 μmol) in TFA (1.0 mL) was stirred at RT for 21 h. The mixture was concentrated in vacuo. The residue was purified using preparative HPLC (column: C30-UG-5, MeCN-H_2_O w/0.1% AcOH = 20 to 70%). The collected fraction was concentrated in vacuo. The resulting aqueous solution was freeze-dried to give PGF00452 (10 mg, 22% yield) as a white powder.

^1^H NMR (300 MHz, CD_3_OD) δ 7.43–7.36 (m, 5H), 7.23–7.14 (m, 5H), 5.75–5.70 (m, 1H), 5.28 (s, 2H), 4.89–4.84 (m, 1H), 4.42–4.41 (m, 1H), 3.77–3.55 (m, 3H), 3.33–3.31 (m, 1H), 3.26–3.13 (m, 2H), 2.90 (t, *J* = 6.0 Hz, 2H), 2.92–2.67 (m, 1H), 2.04–1.97 (m, 1H), 1.82–1.76 (m, 1H), 1.59–1.52 (m, 2H), 1.04 (d, *J* = 9.0 Hz, 3H), 0.87 (d, *J* = 9.0 Hz, 3H).

^13^C NMR (75 MHz, CDCl_3_) δ 165.97, 157.48, 153.95, 137.94, 134.61, 128.95, 128.76, 128.66, 128.38, 126.85, 84.82, 77.24, 69.32, 60.87, 53.74, 50.11, 49.30, 40.20, 33.11, 28.83, 28.32, 24.81, 18.37, 15.88.

HRMS (EI) *m*/*z*: calcd. for C_29_H_38_N_6_O_5_ [M + H]^+^, 551.2982; found, 551.2962.

#### 4.7.5. Synthesis of PGF00478

Benzyl (((*R*)-3-cyclohexyl-1-(((*S*)-1-((2,2-diethoxyethyl)(phenethyl)amino)-1-oxo-5-(3-((2,2,4,6,7-pentamethyl-2,3-dihydrobenzofuran-5-yl)sulfonyl)guanidino)pentan-2-yl)amino)-1-oxopropan-2-yl)oxy)carbamate (**13**).

A mixture of (*R*)-2-((((benzyloxy)carbonyl)amino)oxy)-3-cyclohexylpropanoic acid **12** (50 mg, 0.16 mmol), **9** (0.10 g, 0.16 mmol), and DMT-MM (65 mg, 0.23 mmol) in MeOH (2.0 mL) was stirred at RT for 20 h. The mixture was loaded in an inject column and was purified using column chromatography (SiO_2_, hexane/EtOAc = 1/0 to 0/1, gradient) to give **13** (61 mg, 41% yield) as a colorless oil.

LC/MS *m*/*z* 949.6 [M + H]^+^.

Benzyl (3*R*,6*S*,9a*S*)-3-(cyclohexylmethyl)-4,7-dioxo-6-(3-(3-((2,2,4,6,7-pentamethyl-2,3-dihydrobenzofuran-5-yl)sulfonyl)guanidino)propyl)-8-phenethylhexahydropyrazino [2,1-c][1,2,4]oxadiazine-1(6*H*)-carboxylate (**14**).

A solution of **13** (61 mg, 64 μmol) in formic acid (1.5 mL) was left to stand at RT for 20 h. The mixture was concentrated in vacuo. The residue was purified by column chromatography (SiO_2_, hexane/EtOAc = 1/1 to 0/1, gradient) to give **14** (43 mg, 78% yield) as a colorless amorphous substance.

LC/MS *m*/*z* 858.5 [M + H]^+^.

Benzyl (3*R*,6*S*,9a*S*)-3-(cyclohexylmethyl)-6-(3-guanidinopropyl)-4,7-dioxo-8-phenethylhexahydropyrazino [2,1-c][1,2,4]oxadiazine-1(6*H*)-carboxylate (PGF00478).

A solution of **14** (41 mg, 48 μmol) in TFA (1.0 mL) and water (0.1 mL) was left to stand at RT for 2 h. The mixture was concentrated in vacuo. The residue was purified by preparative HPLC (column: C30-UG-5, MeCN-H_2_O w/0.1% AcOH = 20 to 70%). The collected fraction was concentrated in vacuo. The resulting aqueous solution was freeze-dried to give PGF00478 (22 mg, 76% yield) as a white solid.

^1^H NMR (300 MHz, CD_3_OD) δ 7.42–7.38 (m, 5H), 7.29–7.16 (m, 5H), 5.73 (dd, *J* = 9.0 Hz, 3.0 Hz, 1H), 5.31–5.22 (m, 2H), 4.90–4.84 (m, 1H), 4.57–4.54 (m, 1H), 3.67–3.60 (m, 3H), 3.32–3.14 (m, 2H), 2.93–2.87 (m, 2H), 2.02–1.42 (m, 13H), 1.18–1.11 (m, 3H), 0.94–0.83 (m, 2H).

^13^C NMR (75 MHz, CDCl_3_) δ 179.45, 166.62, 166.01, 157.85, 153.84, 138.04, 134.42, 128.99, 128.81, 128.74, 128.64, 126.82, 79.58, 77.24, 69.49, 60.85, 53.93, 49.71, 49.33, 40.10, 36.78, 33.79, 33.74, 33.08, 31.77, 28.39, 26.28, 26.16, 25.94, 25.07, 24.82.

HRMS (EI) *m*/*z*: calcd. for C_33_H_44_N_6_O_5_ [M + H]^+^, 605.3451; found, 605.3428.

#### 4.7.6. Synthesis of PGF00506

(*S*)-2-amino-*N*-(2,2-diethoxyethyl)-*N*-(3,3-diphenylpropyl)-5-(3-((2,2,4,6,7-pentamethyl-2,3-dihydrobenzofuran-5-yl)sulfonyl)guanidino)pentanamide (**17**).

To a stirring mixture of *N*-(2,2-diethoxyethyl)-3,3-diphenylpropan-1-amine (**15**) (40 g, 123 mmol), **7** (69 g, 123 mmol), and DIEA (43 mL, 264 mmol) in DCM (400 mL) was added HATU (51 g, 135 mmol) in portions at 0 °C. The reaction mixture was stirred at 15 °C for 18 h. The reaction mixture was washed with water (3 × 200 mL), dried over anhydrous Na_2_SO_4_, filtered and concentrated in vacuo. The residue was purified by column chromatography (SiO_2_, petroleum ether/ethyl acetate = 10/1 to 2/1) to give benzyl (*S*)-(1-((2,2-diethoxyethyl)(3,3-diphenylpropyl)amino)-1-oxo-5-(3-((2,2,4,6,7-pentamethyl-2,3-dihydrobenzofuran-5-yl)sulfonyl)guanidino)pentan-2-yl)carbamate (**16**) (60 g, 56% yield) as a yellow oil. A part of this material (30 g, 34 mmol) was dissolved in EtOH (150 mL), and Pd/C (3.0 g, 10% purity) was added under nitrogen atmosphere. The suspension was degassed under vacuum and was purged with hydrogen several times. The mixture was stirred under hydrogen (15 psi) at 25 °C for 72 h. The reaction mixture was filtered. The filtrate was concentrated under reduced pressure to give **17** (10.6 g, 41% yield) as a yellow solid.

LC/MS *m*/*z* 736.3 [M + H]^+^.

Benzyl (((*R*)-3-cyclohexyl-1-(((*S*)-1-((2,2-diethoxyethyl)(3,3-diphenylpropyl)amino)-1-oxo-5-(3-((2,2,4,6,7-pentamethyl-2,3-dihydrobenzofuran-5-yl)sulfonyl)guanidino)pentan-2-yl)amino)-1-oxopropan-2-yl)oxy)carbamate (**18**).

A mixture of **12** (60 mg, 0.19 mmol), **17** (111 mg, 0.15 mmol), DIEA (39 μL, 0.23 mmol), and HATU (63 mg, 0.17 mmol) in DMF (2.0 mL) was stirred at RT for 0.5 h. The mixture was loaded in an inject column and was purified using column chromatography (SiO_2_, hexane/EtOAc = 1/0 to 1/1, gradient) to give **18** (157 mg, 100% yield) as a colorless oil.

LC/MS *m*/*z* 1040.6 [M + H]^+^.

Benzyl (3*R*,6*S*,9a*S*)-3-(cyclohexylmethyl)-8-(3,3-diphenylpropyl)-4,7-dioxo-6-(3-(3-((2,2,4,6,7-pentamethyl-2,3-dihydrobenzofuran-5-yl)sulfonyl)guanidino)propyl)hexahydropyrazino [2,1-c][1,2,4]oxadiazine-1(6*H*)-carboxylate (**19**).

A mixture of **18** (157 mg, 0.15 mmol) and formic acid (2.0 mL) was left to stand at RT for 20 h, and then at 60 °C for 3 h. After cooling to RT, the mixture was concentrated in vacuo to give **19** (143 mg, 100% yield) as a pale brown oil. This material was used in the next step without purification.

LC/MS *m*/*z* 947.5 [M + H]^+^.

Benzyl (3*R*,6*S*,9a*S*)-3-(cyclohexylmethyl)-8-(3,3-diphenylpropyl)-6-(3-guanidinopropyl)-4,7-dioxohexahydropyrazino [2,1-c][1,2,4]oxadiazine-1(6*H*)-carboxylate (PGF00506).

A solution of **19** (143 mg, 0.15 mmol) in TFA (1.0 mL) and water (0.1 mL) was stirred at RT for 2 h, and then at 60 °C for 2 h. To the mixture was added TFA (2.0 mL). The mixture was stirred at 60 °C for 1.5 h. After cooling to RT, the mixture was concentrated in vacuo. The residue was purified by preparative HPLC (column: C30-UG-5, MeCN-H_2_O w/0.1% AcOH = 20 to 70%). The collected fraction was concentrated in vacuo. The aqueous solution was freeze-dried to give PGF00506 (11 mg, 16 μmol, 11% yield) as a white solid.

^1^H NMR (300 MHz, CD_3_OD) δ 7.43–7.36 (m, 5H), 7.31–7.11 (m, 10H), 5.74 (dd, *J* = 9.0 Hz, 3.0 Hz, 1H), 5.33–5.25 (m, 2H), 4.86–4.81 (m, 1H), 4.57 (dd, *J* = 9.0 Hz, 3.0 Hz, 1H), 3.93 (t, *J* = 9.0 Hz, 1H), 3.78 (t, *J* = 12.0 Hz, 1H), 3.43–3.38 (m, 3H), 3.24–3.16 (m, 2H), 2.39–2.36 (m, 2H), 1.95–1.86 (m, 2H), 1.77–1.42 (m, 10H), 1.16–1.10 (m, 3H), 0.93–0.75 (m, 2H).

^13^C NMR (75 MHz, CDCl_3_) δ 179.29, 166.56, 165.87, 157.91, 153.89, 143.87, 143.85, 134.42, 129.01, 128.82, 128.69, 128.66, 127.53, 126.57, 79.76, 77.23, 70.56, 69.51, 61.01, 54.03, 49.26, 49.10, 47.19, 40.07, 36.72, 33.84, 33.73, 32.37, 31.82, 28.49, 26.28, 26.18, 25.96, 25.17, 24.75.

HRMS (EI) *m*/*z*: calcd. for C_40_H_50_N_6_O_5_ [M + H]^+^, 695.3921; found, 695.3896.

### 4.8. Molecular Docking

To examine the binding interactions of PGF00432 and PGF00506 against CTLA-4, molecular docking was performed using AutoDock Vina [20], as implemented in YASARA [21]. The docking region on CTLA-4 was derived from the crystal structure of B7-1/CTLA-4 complex (PDB code: 1I8L) [22]. B7-1 binds to CTLA-4 in a rigid-body-like manner. The Cα differences between complexed and apo-B7-1 were small (RMS deviation = 0.80 Å) [23]. The only marked change was that Arg94 in the FG loop of B7-1 moved 1–1.5 Å to form an H-bond with Tyr104 of CTLA-4 [23]. Furthermore, Arg94 was located near the center of the binding interface between B7-1 and CTLA-4; consequently, it might be an amino acid that is important for binding to CTLA-4. For this study, a cube with a length of 19.74 Å on each side centered on Arg94 was defined as the docking region on CTLA-4 (Figure 8A). The docking region covered most of the binding interface. In the molecular docking, a macro file (dock_run.mcr) with default parameters in YASARA was used. After molecular docking, a binding interaction model with a score of rank 1 was selected.

## 5. Conclusions

In this study, we succeeded in obtaining peptidemimetic small-molecule compounds that inhibit PPI between CTLA-4 and B7-1 without relying on structural analysis such as X-ray crystallography. The combination of PURE*frex*^®^RD and sequence mimic by a PepMetics^®^ scaffold is anticipated to be a powerful approach for drug discovery targeting PPI inhibition by small molecules.

## Data Availability

Data are contained within the article and Supplementary Materials.

## Supplementary Materials

The following supporting information can be downloaded at https://www.mdpi.com/article/10.3390/ph15121506/s1. Figure S1: Identification of amino acids involved in binding with CTLA-4 by alanine scanning mutants. Figure S2: Acceptable amino acid types in positions not involved in binding with CTLA-4. Figure S3: Frequency of occurrence of amino acids in positions not involved in binding with CTLA-4. Figure S4: 1H NMR spectrum of PGF00432. Figure S5: 13C NMR spectrum of PGF00432. Figure S6: HRMS spectrum of PGF00432. Figure S7: 1H NMR spectrum of PGF00452. Figure S8: 13C NMR spectrum of PGF00452. Figure S9: HRMS spectrum of PGF00452. Figure S10: 1H NMR spectrum of PGF00478. Figure S11: 13C NMR spectrum of PGF00478. Figure S12: HRMS spectrum of PGF00478. Figure S13: 1H NMR spectrum of PGF00506. Figure S14: 13C NMR spectrum of PGF00506. Figure S15: HRMS spectrum of PGF00506.
