# Supplementary material for: Rational Strategy for Designing Peptidomimetic Small Molecules Based on Cyclic Peptides Targeting Protein–Protein Interaction between CTLA-4 and B7-1
Source: Pharmaceuticals (Basel). 2022 Dec 2;15(12):1506. doi: 10.3390/ph15121506 (PMC9784018; doi:10.3390/ph15121506)
Supplement: Supplementary file 1 [file pharmaceuticals-15-01506-s001.zip › pharmaceuticals-2044562-supplementary.pdf]

Supplementary Materials

Rational Strategy for Designing Peptidomimetic Small Molecules Based on Cyclic Peptides Targeting Protein–Protein Interaction between CTLA-4 and B7-1

Kumiko Tsuihiji [1,*], Eiji Honda [2], Kanehisa Kojoh [1], Shizue Katoh [1], Tomonori Taguri [2], Atsushi Yoshimori [3] and Hajime Takashima [2,*]

[1] Drug Discovery Division, GeneFrontier Corporation, SHARP Kashiwa Building, 4F 273-1 Kashiwa, Kashiwa-shi 277-0005, Chiba, Japan; kojoh@genefrontier.com (K.K.); katoh@genefrontier.com (S.K.)

[2] PRISM BioLab Co., Ltd., C21F-4110, 26-1 Muraoka-Higashi 2-Chome, Fujisawa 251-8555, Kanagawa, Japan; e.mmk.honda@gmail.com (E.H.); taguri@prismbiolab.com (T.T.)

[3] Chemoinformatics & AI Research Group, Institute for Theoretical Medicine, Inc.,
BW3M-20B, 26-1 Muraoka-Higashi 2-Chome, Fujisawa 251-0012, Kanagawa, Japan;
yoshimori@itmol.com

* Correspondence: tsuihiji@genefrontier.com (K.T.); takashima@prismbiolab.com (H.T.)

Supplementary Figure S1. Identification of amino acids involved in binding with CTLA-4 by alanine scanning mutants.

Supplementary Figure S2. Acceptable amino acid types in positions not involved in binding with CTLA-4.

Supplementary Figure S3. Frequency of occurrence of amino acids in positions not involved in binding with CTLA-4.

Supplementary Figure S4. ^{1}H NMR spectrum of **PGF00432**.

Supplementary Figure S5. ^{13}C NMR spectrum of **PGF00432**.

Supplementary Figure S6. HRMS spectrum of **PGF00432**.

Supplementary Figure S7. ^{1}H NMR spectrum of **PGF00452**.

Supplementary Figure S8. ^{13}C NMR spectrum of **PGF00452**.

Supplementary Figure S9. HRMS spectrum of **PGF00452**.

Supplementary Figure S10. ^{1}H NMR spectrum of **PGF00478**.

Supplementary Figure S11. ^{13}C NMR spectrum of **PGF00478**.

Supplementary Figure S12. HRMS spectrum of **PGF00478**.

Supplementary Figure S13. ^{1}H NMR spectrum of **PGF00506**.

Supplementary Figure S14. ^{13}C NMR spectrum of **PGF00506**.

Supplementary Figure S15. HRMS spectrum of **PGF00506**.

A

mutant name	linker		Cys	Random loop												Cys	linker	
	−2	−1	Cys	1	2	3	4	5	6	7	8	9	10	11	12	Cys	+1	+2
C4m-310044	G	G	C	L	H	P	F	L	P	I	V	S	H	H	F	C	G	R
L1A	G	G	C	A	H	P	F	L	P	I	V	S	H	H	F	C	G	R
H2A	G	G	C	L	A	P	F	L	P	I	V	S	H	H	F	C	G	R
P3A	G	G	C	L	H	A	F	L	P	I	V	S	H	H	F	C	G	R
F4A	G	G	C	L	H	P	A	L	P	I	V	S	H	H	F	C	G	R
L5A	G	G	C	L	H	P	F	A	P	I	V	S	H	H	F	C	G	R
P6A	G	G	C	L	H	P	F	L	A	I	V	S	H	H	F	C	G	R
I7A	G	G	C	L	H	P	F	L	P	A	V	S	H	H	F	C	G	R
V8A	G	G	C	L	H	P	F	L	P	I	A	S	H	H	F	C	G	R
S9A	G	G	C	L	H	P	F	L	P	I	V	A	H	H	F	C	G	R
H10A	G	G	C	L	H	P	F	L	P	I	V	S	A	H	F	C	G	R
H11A	G	G	C	L	H	P	F	L	P	I	V	S	H	A	F	C	G	R
F12A	G	G	C	L	H	P	F	L	P	I	V	S	H	H	A	C	G	R
G(+1)A	G	G	C	L	H	P	F	L	P	I	V	S	H	H	F	C	A	R
R(+2)A	G	G	C	L	H	P	F	L	P	I	V	S	H	H	F	C	G	A

B

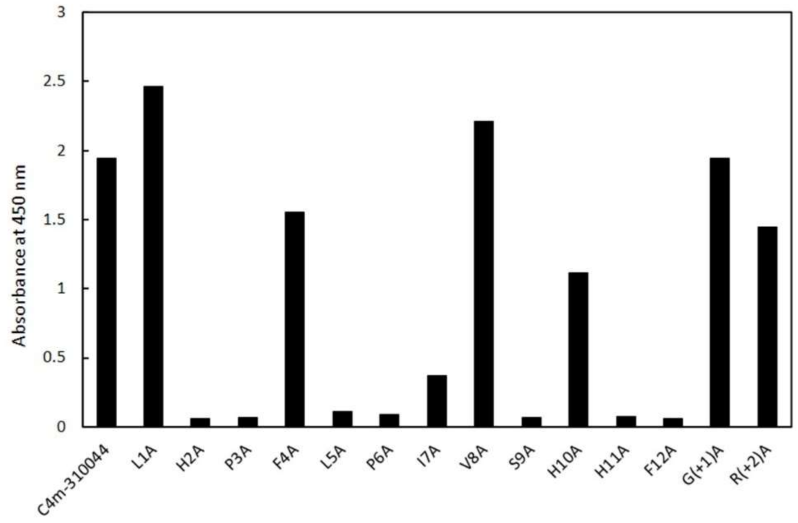

Supplementary Figure S1. Identification of amino acids involved in binding with CTLA-4 by alanine scanning mutants.

(A) List of alanine scanning mutants based on C4m-310044 with affinity as high as that of C4m-3127. Alanine substitutions were generated via site-directed mutagenesis, employing a pET-based vector to express the MBP-peptide fusion protein as the expression plasmid, the corresponding primer sets, and KOD plus DNA polymerase, to ascertain which residues are involved in interaction with CTLA-4. All mutations were confirmed using DNA sequence

analysis. (B) 14 variants shown in (A) were expressed as the MBP-peptide fusion protein. The binding activity of each on CTLA-4 was evaluated using lysate ELISA. The BL21(DE3) *E. coli* cells transformed with each alanine scanning mutant were grown in 100 µL of 2xYT/50 µg/mL ampicillin at 37 °C until OD600 of 0.5–0.8 was reached. After IPTG (0.5 mM) was added, the broth was incubated at 25 °C, with shaking overnight. The cell broth was lysed using lysis buffer (5 mg/mL lysozyme (FUJIFILM Wako) in PBS, 50% BugBuster® (v/v) (Merck Millipore) at RT for 2 h, with shaking. The lysate was blocked with TBS/0.05% Tween-20/12.5% skim milk by incubating at RT for 0.5 h, shaking, then added to CTLA-4-immobilized wells on 384-plate and incubated at RT for 1.5 h. After washing and incubation with anti-FLAG M2®-peroxidase antibody (1:10,000; Sigma Aldrich) at RT for 1 h, the absorbance at 450 nm/ 650 nm was read with a microplate reader through the reaction with 1x TMB substrate solution and 2N hydrochloric acid.

mutant name	linker		Cys	(NNS)	H	P	(NNS)	L	P	I	(NNS)	S	(NNS)	H	F	Cys	(NNS)	(NNS)	ELISA	EC50
	−2	−1	0	1	2	3	4	5	6	7	8	9	10	11	12	0	+1	+2	S/N	(μM)
5th4w-NN6-67	G	G	C	L	H	P	F	L	P	I	R	S	V	H	F	C	P	R	45.4	0.0257
5th4w-NN6-15	G	G	C	L	H	P	F	L	P	I	R	S	V	H	F	C	P	F	39.9	
5th4w-NN6-25	G	G	C	L	H	P	V	L	P	I	R	S	R	H	F	C	P	V	38.9	
5th4w-NN6-21	G	G	C	W	H	P	W	L	P	I	L	S	P	H	F	C	P	R	37.5	0.0455
5th4w-NN6-39	G	G	C	W	H	P	F	L	P	I	R	S	L	H	F	C	P	R	35.6	
5th4w-NN6-70	G	G	C	M	H	P	F	L	P	I	R	S	M	H	F	C	P	L	35.5	
5th2w-NNS6-14	G	G	C	W	H	P	F	L	P	I	R	S	Y	H	F	C	P	W	34.7	
5th4w-NN6-47	G	G	C	P	H	P	W	L	P	I	F	S	R	H	F	C	P	V	34.2	
5th4w-NN6-54	G	G	C	L	H	P	G	L	P	I	R	S	L	H	F	C	P	R	33.3	
5th4w-NN6-81	G	G	C	F	H	P	Y	L	P	I	R	S	W	H	F	C	S	R	32.3	
5th4w-NN6-53	G	G	C	R	H	P	F	L	P	I	Y	S	V	H	F	C	A	C	32.2	
5th4w-NN6-34	G	G	C	F	H	P	F	L	P	I	R	S	D	H	F	C	S	R	32.2	
5th4w-NN6-4	G	G	C	R	H	P	Y	L	P	I	V	S	R	H	F	C	P	F	31.6	
5th2w-NNS6-21	G	G	C	W	H	P	F	L	P	I	R	S	S	H	F	C	P	S	31.1	
5th4w-NN6-20	G	G	C	S	H	P	Q	L	P	I	L	S	D	H	F	C	S	A	30.6	
5th4w-NN6-77	G	G	C	R	H	P	F	L	P	I	V	S	T	H	F	C	S	R	30.5	
5th4w-NN6-23	G	G	C	R	H	P	H	L	P	I	W	S	W	H	F	C	G	R	30.2	0.0476
5th4w-NN6-44	G	G	C	R	H	P	F	L	P	I	V	S	W	H	F	C	G	R	30.1	
5th4w-NN6-92-1	G	G	C	V	H	P	F	L	P	I	R	S	V	H	F	C	A	L	30.0	
5th4w-NN6-19	G	G	C	R	H	P	Y	L	P	I	W	S	I	H	F	C	Q	K	29.7	
5th4w-NN6-55-1	G	G	C	W	H	P	F	L	P	I	S	S	P	H	F	C	G	R	29.5	
5th4w-NN6-55-4	G	G	C	V	H	P	S	L	P	I	L	S	V	H	F	C	P	R	29.5	
5th2w-NNS6-22	G	G	C	M	H	P	F	L	P	I	Q	S	V	H	F	C	S	R	29.3	
5th4w-NN6-93	G	G	C	C	H	P	W	L	P	I	V	S	T	H	F	C	G	L	28.8	
5th4w-NN6-9	G	G	C	F	H	P	Y	L	P	I	R	S	W	H	F	C	G	R	28.8	
5th4w-NN6-52	G	G	C	L	H	P	F	L	P	I	R	S	V	H	F	C	G	C	28.6	
5th2w-NNS6-55	G	G	C	M	H	P	Y	L	P	I	K	S	V	H	F	C	P	L	28.3	
5th4w-NN6-82	G	G	C	F	H	P	F	L	P	I	R	S	L	H	F	C	C	R	28.3	
5th4w-NN6-50	G	G	C	K	H	P	V	L	P	I	W	S	W	H	F	C	R	I	28.1	
5th4w-NN6-78	G	G	C	W	H	P	A	L	P	I	R	S	W	H	F	C	P	R	27.9	0.0277
5th4w-NN6-57	G	G	C	M	H	P	Y	L	P	I	V	S	V	H	F	C	P	C	27.7	
5th4w-NN6-59	G	G	C	L	H	P	H	L	P	I	W	S	L	H	F	C	P	L	27.6	
5th4w-NN6-24	G	G	C	W	H	P	W	L	P	I	Y	S	H	H	F	C	P	I	27.6	
5th4w-NN6-32	G	G	C	P	H	P	F	L	P	I	W	S	V	H	F	C	P	F	27.2	
5th4w-NN6-56	G	G	C	W	H	P	F	L	P	I	Q	S	L	H	F	C	G	V	27.1	
5th2w-NNS6-67	G	G	C	Q	H	P	W	L	P	I	S	S	W	H	F	C	P	R	25.5	
5th4w-NN6-69-1	G	G	C	S	H	P	F	L	P	I	L	S	V	H	F	C	P	F	24.9	
5th4w-NN6-69-3	G	G	C	L	H	P	W	L	P	I	V	S	V	H	F	C	A	R	24.9	
5th4w-NN6-79	G	G	C	W	H	P	V	L	P	I	R	S	C	H	F	C	A	V	24.9	
5th4w-NN6-68	G	G	C	L	H	P	W	L	P	I	R	S	R	H	F	C	S	R	24.7	
4th-NNS6-28	G	G	C	L	H	P	F	L	P	I	F	S	S	H	F	C	A	L	23.2	
5th4w-NN6-7	G	G	C	R	H	P	F	L	P	I	W	S	W	H	F	C	P	F	23.0	
5th4w-NN6-85	G	G	C	W	H	P	F	L	P	I	F	S	V	H	F	C	G	C	22.9	
5th2w-NNS6-71	G	G	C	R	H	P	Y	L	P	I	A	S	P	H	F	C	P	G	22.9	
4th-NNS6-90	G	G	C	K	H	P	V	L	P	I	V	S	L	H	F	C	P	W	22.9	

5th4w-NN6-37	G	G	C	L	H	P	F	L	P	I	S	S	W	H	F	C	G	R	22.7	
5th2w-NNS6-94	G	G	C	F	H	P	Y	L	P	I	S	S	V	H	F	C	G	R	22.4	
5th4w-NN6-51	G	G	C	W	H	P	Y	L	P	I	V	S	W	H	F	C	S	R	22.1	
5th4w-NN6-11	G	G	C	R	H	P	F	L	P	I	W	S	H	H	F	C	V	C	22.1	
5th4w-NN6-87	G	G	C	L	H	P	V	L	P	I	R	S	F	H	F	C	W	R	21.4	
4th-NNS6-87	G	G	C	R	H	P	W	L	P	I	V	S	W	H	F	C	S	R	21.4	
5th4w-NN6-90	G	G	C	R	H	P	W	L	P	I	V	S	Y	H	F	C	S	S	21.2	
5th2w-NNS6-25	G	G	C	K	H	P	F	L	P	I	S	S	H	H	F	C	P	Y	21.1	
4th-NNS6-83	G	G	C	R	H	P	F	L	P	I	F	S	K	H	F	C	E	L	20.7	
4th-NNS6-17	G	G	C	R	H	P	V	L	P	I	V	S	L	H	F	C	P	G	20.4	
5th2w-NNS6-12	G	G	C	W	H	P	Y	L	P	I	R	S	V	H	F	C	G	R	20.4	
4th-NNS6-72	G	G	C	V	H	P	Y	L	P	I	S	S	I	H	F	C	S	M	20.0	
5th2w-NNS6-90	G	G	C	R	H	P	F	L	P	I	V	S	F	H	F	C	P	F	19.7	
5th4w-NN6-86	G	G	C	A	H	P	V	L	P	I	R	S	L	H	F	C	P	H	19.7	
5th4w-NN6-58	G	G	C	R	H	P	F	L	P	I	S	S	I	H	F	C	C	I	18.7	
5th2w-NNS6-3	G	G	C	A	H	P	F	L	P	I	F	S	P	H	F	C	P	F	18.4	
5th4w-NN6-2	G	G	C	W	H	P	H	L	P	I	V	S	S	H	F	C	L	F	18.4	
5th4w-NN6-71	G	G	C	P	H	P	W	L	P	I	A	S	V	H	F	C	P	F	17.4	
5th4w-NN6-62	G	G	C	Q	H	P	Y	L	P	I	W	S	F	H	F	C	P	F	17.2	0.135
5th4w-NN6-30	G	G	C	W	H	P	F	L	P	I	M	S	F	H	F	C	P	Y	16.7	
5th4w-NN6-38	G	G	C	S	H	P	F	L	P	I	F	S	L	H	F	C	P	V	16.6	
5th2w-NNS6-8	G	G	C	R	H	P	F	L	P	I	F	S	L	H	F	C	A	R	16.5	
4th-NNS6-16	G	G	C	W	H	P	Y	L	P	I	F	S	V	H	F	C	P	R	16.5	
5th4w-NN6-63	G	G	C	W	H	P	Y	L	P	I	L	S	R	H	F	C	Q	A	16.5	
5th4w-NN6-73	G	G	C	R	H	P	W	L	P	I	S	S	A	H	F	C	R	R	16.2	
5th4w-NN6-14	G	G	C	W	H	P	F	L	P	I	R	S	V	H	F	C	C	R	16.1	
5th4w-NN6-1	G	G	C	R	H	P	Y	L	P	I	F	S	A	H	F	C	R	S	16.1	
5th4w-NN6-6	G	G	C	M	H	P	F	L	P	I	K	S	S	H	F	C	R	K	16.1	
5th2w-NNS6-51	G	G	C	R	H	P	F	L	P	I	V	S	L	H	F	C	P	F	15.8	
5th4w-NN6-45	G	G	C	R	H	P	W	L	P	I	W	S	V	H	F	C	G	V	15.8	
4th-NNS6-12	G	G	C	S	H	P	F	L	P	I	R	S	R	H	F	C	P	F	15.8	
5th2w-NNS6-75	G	G	C	W	H	P	W	L	P	I	K	S	P	H	F	C	A	L	15.3	
5th4w-NN6-74	G	G	C	F	H	P	F	L	P	I	Y	S	W	H	F	C	A	R	15.1	
5th4w-NN6-5-1	G	G	C	W	H	P	Y	L	P	I	Q	S	L	H	F	C	P	F	14.7	
5th4w-NN6-5-3	G	G	C	W	H	P	F	L	P	I	L	S	L	H	F	C	G	W	14.7	
5th4w-NN6-46	G	G	C	V	H	P	A	L	P	I	W	S	F	H	F	C	P	S	14.2	
5th4w-NN6-64	G	G	C	W	H	P	V	L	P	I	R	S	V	H	F	C	C	R	13.7	
4th-NNS6-48	G	G	C	F	H	P	W	L	P	I	A	S	W	H	F	C	G	R	13.5	
4th-NNS6-64	G	G	C	L	H	P	Y	L	P	I	V	S	S	H	F	C	P	F	12.9	
4th-NNS6-80	G	G	C	G	H	P	F	L	P	I	F	S	L	H	F	C	P	R	12.8	
4th-NNS6-79	G	G	C	L	H	P	F	L	P	I	R	S	V	H	F	C	L	R	12.8	
4th-NNS6-2	G	G	C	W	H	P	S	L	P	I	Y	S	P	H	F	C	P	L	12.8	
5th4w-NN6-65	G	G	C	W	H	P	F	L	P	I	L	S	L	H	F	C	S	R	12.5	
5th4w-NN6-83	G	G	C	L	H	P	L	L	P	I	L	S	V	H	F	C	P	S	12.0	
4th-NNS6-60	G	G	C	W	H	P	F	L	P	I	S	S	W	H	F	C	G	L	11.9	
5th2w-NNS6-53	G	G	C	L	H	P	W	L	P	I	R	S	F	H	F	C	G	A	11.4	
5th2w-NNS6-45	G	G	C	R	H	P	F	L	P	I	W	S	V	H	F	C	W	R	11.0	
4th-NNS6-3	G	G	C	S	H	P	F	L	P	I	W	S	V	H	F	C	G	Q	10.9	

5th2w-NNS6-6	G	G	C	W	H	P	Y	L	P	I	L	S	L	H	F	C	Q	R	10.3
5th2w-NNS6-27	G	G	C	F	H	P	F	L	P	I	R	S	C	H	F	C	A	R	9.9
5th2w-NNS6-37	G	G	C	I	H	P	F	L	P	I	W	S	I	H	F	C	S	R	9.4
5th2w-NNS6-33	G	G	C	R	H	P	F	L	P	I	W	S	F	H	F	C	W	G	9.4
5th2w-NNS6-79	G	G	C	R	H	P	F	L	P	I	V	S	R	H	F	C	V	A	9.2
4th-NNS6-85	G	G	C	G	H	P	F	L	P	I	F	S	S	H	F	C	P	L	9.1
5th2w-NNS6-30	G	G	C	W	H	P	F	L	P	I	L	S	W	H	F	C	G	R	9.0
5th4w-NN6-89	G	G	C	M	H	P	F	L	P	I	I	S	Q	H	F	C	A	F	8.3
5th2w-NNS6-86	G	G	C	F	H	P	Y	L	P	I	W	S	L	H	F	C	R	S	7.7
4th-NNS6-30	G	G	C	W	H	P	C	L	P	I	L	S	P	H	F	C	P	R	7.5
4th-NNS6-26	G	G	C	A	H	P	A	L	P	I	W	S	C	H	F	C	P	F	7.1
5th2w-NNS6-74	G	G	C	M	H	P	F	L	P	I	R	S	W	H	F	C	S	R	7.0
4th-NNS6-89	G	G	C	W	H	P	F	L	P	I	F	S	A	H	F	C	M	R	6.6
5th2w-NNS6-84	G	G	C	W	H	P	F	L	P	I	V	S	F	H	F	C	P	F	6.4
5th2w-NNS6-28	G	G	C	A	H	P	Y	L	P	I	R	S	V	H	F	C	P	C	6.3
5th2w-NNS6-4	G	G	C	F	H	P	W	L	P	I	S	S	L	H	F	C	P	F	6.3
5th2w-NNS6-46	G	G	C	F	H	P	F	L	P	I	F	S	Y	H	F	C	G	L	6.1
5th4w-NN6-61	G	G	C	P	H	P	M	L	P	I	F	S	V	H	F	C	P	Y	5.9
4th-NNS6-94	G	G	C	R	H	P	V	L	P	I	W	S	S	H	F	C	P	W	5.9
5th2w-NNS6-26	G	G	C	L	H	P	F	L	P	I	V	S	I	H	F	C	S	A	5.8
5th4w-NN6-36	G	G	C	R	H	P	Y	L	P	I	W	S	A	H	F	C	C	R	5.7
5th4w-NN6-75	G	G	C	W	H	P	F	L	P	I	K	S	V	H	F	C	C	A	5.6
5th2w-NNS6-70	G	G	C	R	H	P	F	L	P	I	V	S	W	H	F	C	A	A	5.1
5th2w-NNS6-34	G	G	C	C	H	P	F	L	P	I	Y	S	V	H	F	C	A	S	5.0
5th4w-NN6-94	G	G	C	L	H	P	F	L	P	I	Y	S	L	H	F	C	P	C	5.0
4th-NNS6-66	G	G	C	A	H	P	H	L	P	I	R	S	V	H	F	C	S	F	4.8
5th2w-NNS6-43	G	G	C	R	H	P	F	L	P	I	W	S	S	H	F	C	A	A	4.8
4th-NNS6-7	G	G	C	R	H	P	F	L	P	I	R	S	P	H	F	C	G	S	4.7
5th4w-NN6-72	G	G	C	W	H	P	W	L	P	I	V	S	V	H	F	C	G	C	4.6
4th-NNS6-70	G	G	C	S	H	P	Y	L	P	I	H	S	R	H	F	C	L	S	4.6
5th2w-NNS6-68	G	G	C	L	H	P	W	L	P	I	V	S	C	H	F	C	P	R	4.6
5th2w-NNS6-87	G	G	C	L	H	P	Y	L	P	I	F	S	V	H	F	C	A	L	4.5
5th2w-NNS6-66	G	G	C	R	H	P	F	L	P	I	F	S	V	H	F	C	P	F	4.4
4th-NNS6-20	G	G	C	L	H	P	F	L	P	I	L	S	L	H	F	C	P	F	4.4
5th2w-NNS6-61	G	G	C	C	H	P	W	L	P	I	R	S	V	H	F	C	P	F	4.3
4th-NNS6-19	G	G	C	M	H	P	S	L	P	I	W	S	W	H	F	C	P	H	4.1
5th4w-NN6-40	G	G	C	F	H	P	F	L	P	I	R	S	H	H	F	C	M	L	4.0
5th4w-NN6-49	G	G	C	M	H	P	Y	L	P	I	R	S	F	H	F	C	S	C	4.0
5th4w-NN6-80	G	G	C	R	H	P	F	L	P	I	F	S	W	H	F	C	A	V	3.8
5th2w-NNS6-80	G	G	C	L	H	P	F	L	P	I	V	S	W	H	F	C	P	C	3.6
4th-NNS6-37	G	G	C	W	H	P	V	L	P	I	R	S	I	H	F	C	G	C	3.5
5th2w-NNS6-93	G	G	C	F	H	P	F	L	P	I	Q	S	R	H	F	C	C	R	3.4
4th-NNS6-95	G	G	C	W	H	P	V	L	P	I	R	S	G	H	F	C	S	R	3.4
5th2w-NNS6-41	G	G	C	W	H	P	V	L	P	I	R	S	D	H	F	C	C	R	3.1

Supplementary Figure S2. Acceptable amino acid types in positions not involved in binding with CTLA-4. Residues highlighted in gray indicate mutated sites.

Types of	Cys	(NNS)	H	P	(NNS)	L	P	I	(NNS)	S	(NNS)	H	F	Cys	(NNS)	(NNS)	Total
amino acid	0	1	2	3	4	5	6	7	8	9	10	11	12	0	+1	+2	
W		34			19				20		20				3	4	100
F		13			67				17		9				0	22	128
Y		0			25				6		3				0	3	37
V		4			12				22		33				2	7	80
L		21			1				12		20				3	14	71
I		1			0				2		6				0	3	12
M		10			1				1		1				2	1	16
P		4			0				0		9				56	0	69
A		5			3				3		4				15	8	38
G		2			1				0		1				22	4	30
S		6			3				10		8				17	9	53
T		0			0				0		2				0	0	2
N		0			0				0		0				0	0	0
Q		2			1				4		1				3	1	12
D		0			0				0		3				0	0	3
E		0			0				0		0				1	0	1
K		3			0				4		1				0	2	10
R		30			0				36		9				6	47	128
H		0			4				1		4				0	2	11
C		3			1				0		4				8	11	27

Supplementary Figure S3. Frequency of occurrence of amino acids in positions not involved in binding with CTLA-4.

The frequency of the amino acids with aromatic group (Trp, Phe, Tyr), hydrophobic group (Val, Leu), and long side chain (Arg) was high in the loop of the cyclic peptide. By contrast, the frequency of the amino acids with small side chains (Pro, Ala, Gly, Ser) was high in the position immediately adjacent to the C-terminal cysteine.

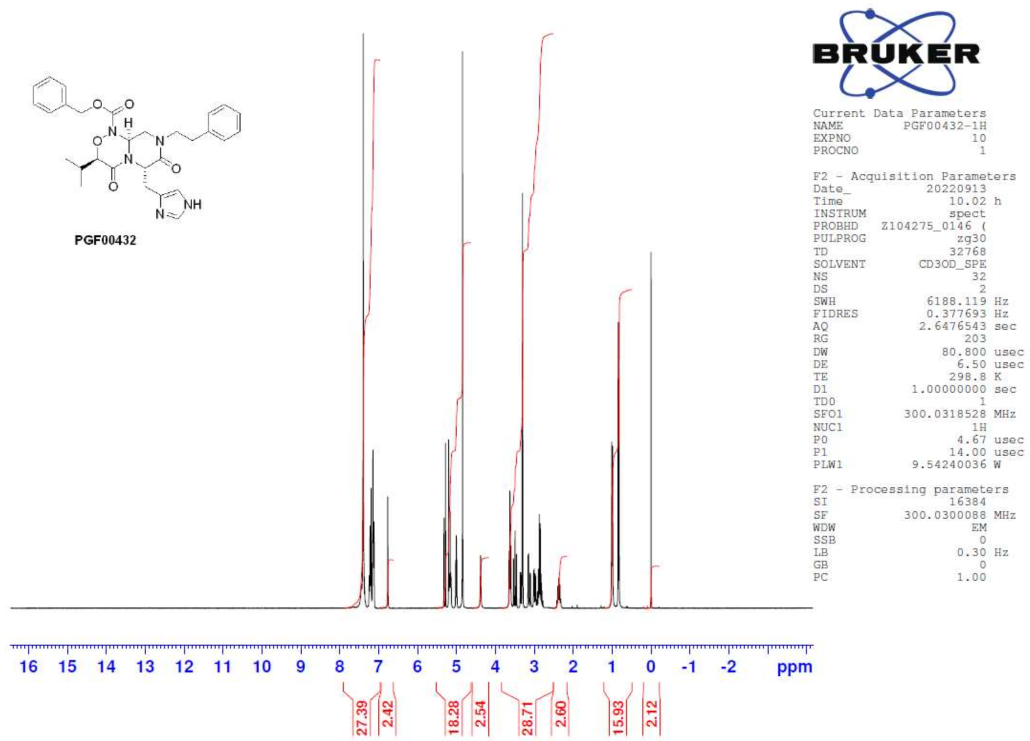

Supplementary Figure S4. ^{1}H NMR spectrum of **PGF00432**.

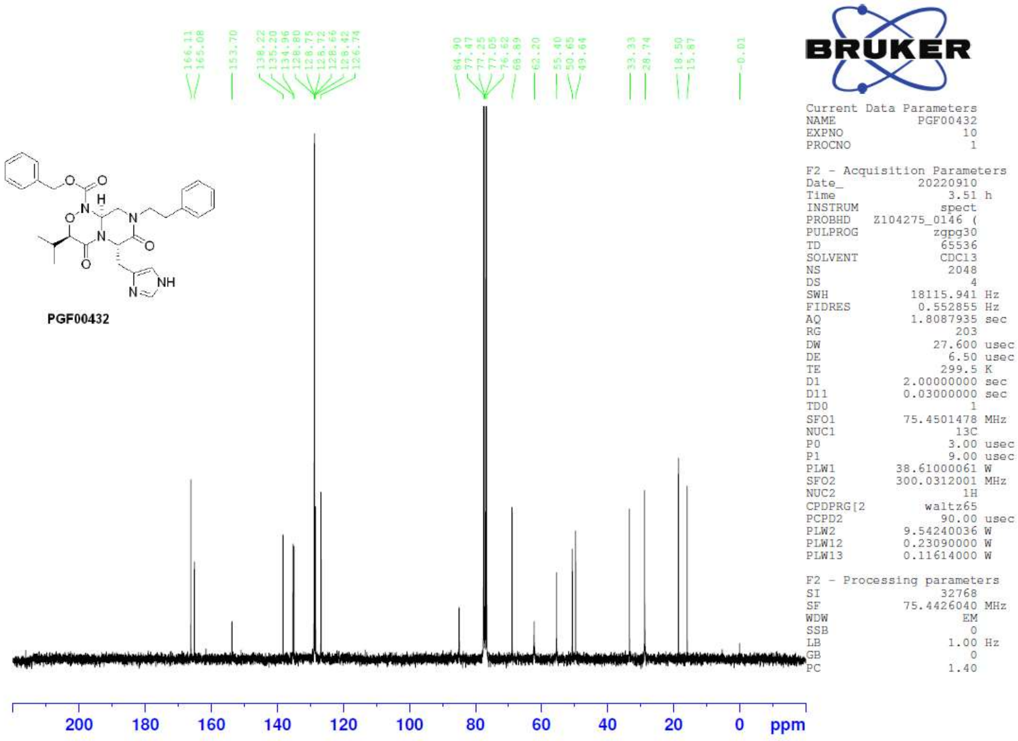

Supplementary Figure S5. ^{13}C NMR spectrum of **PGF00432**.

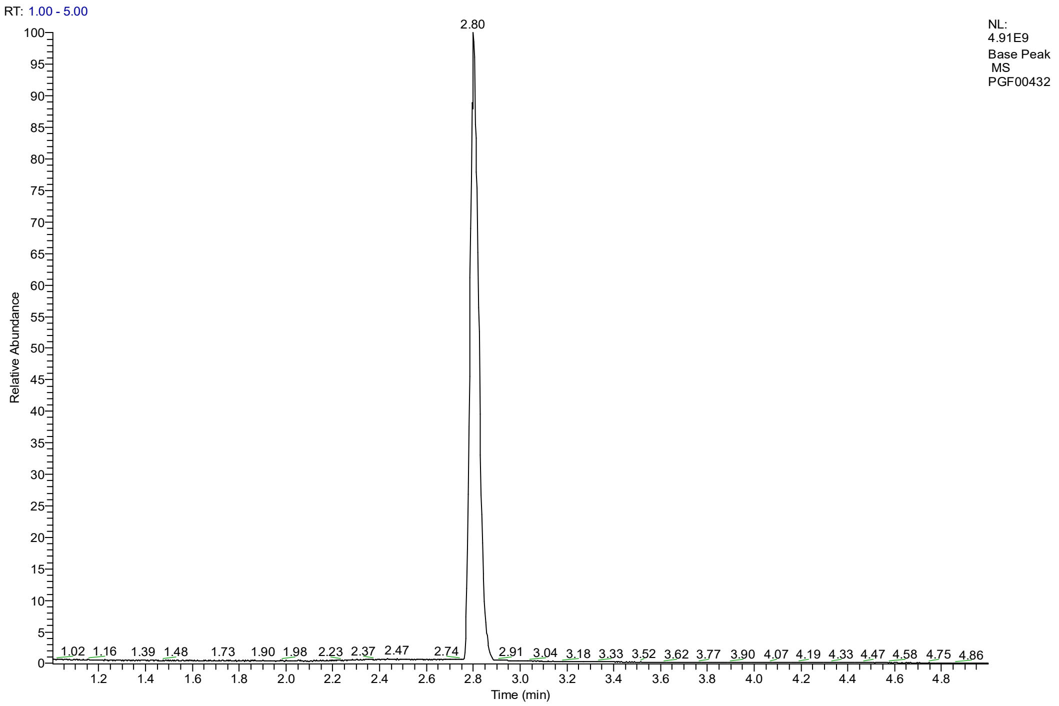

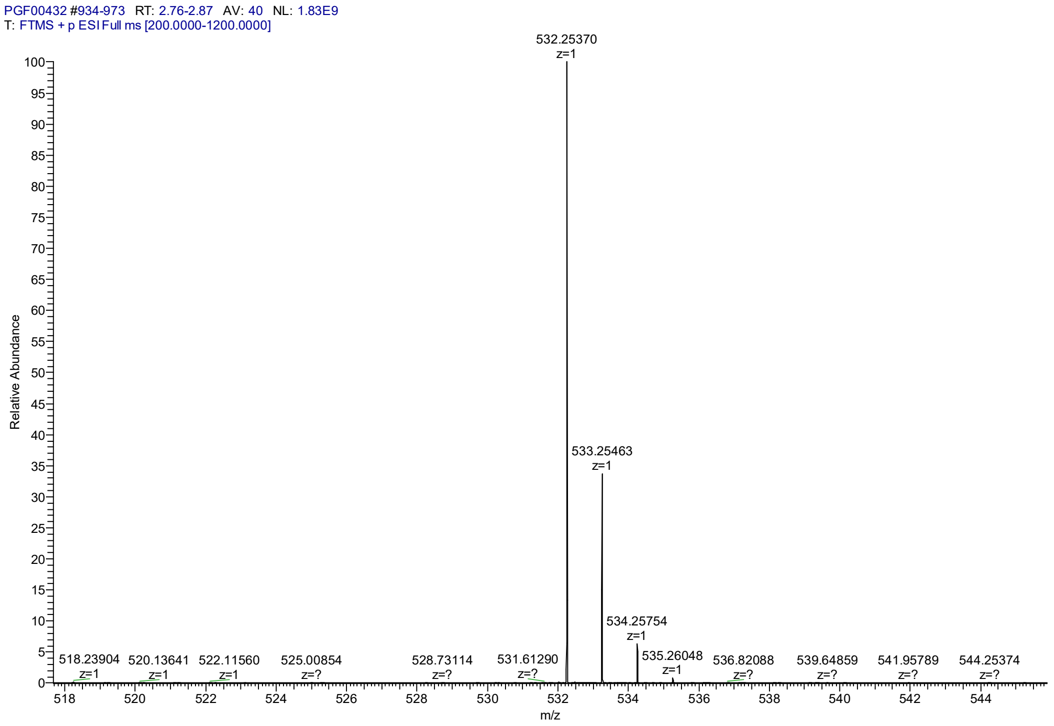

Supplementary Figure S6. HRMS spectrum of **PGF00432**.

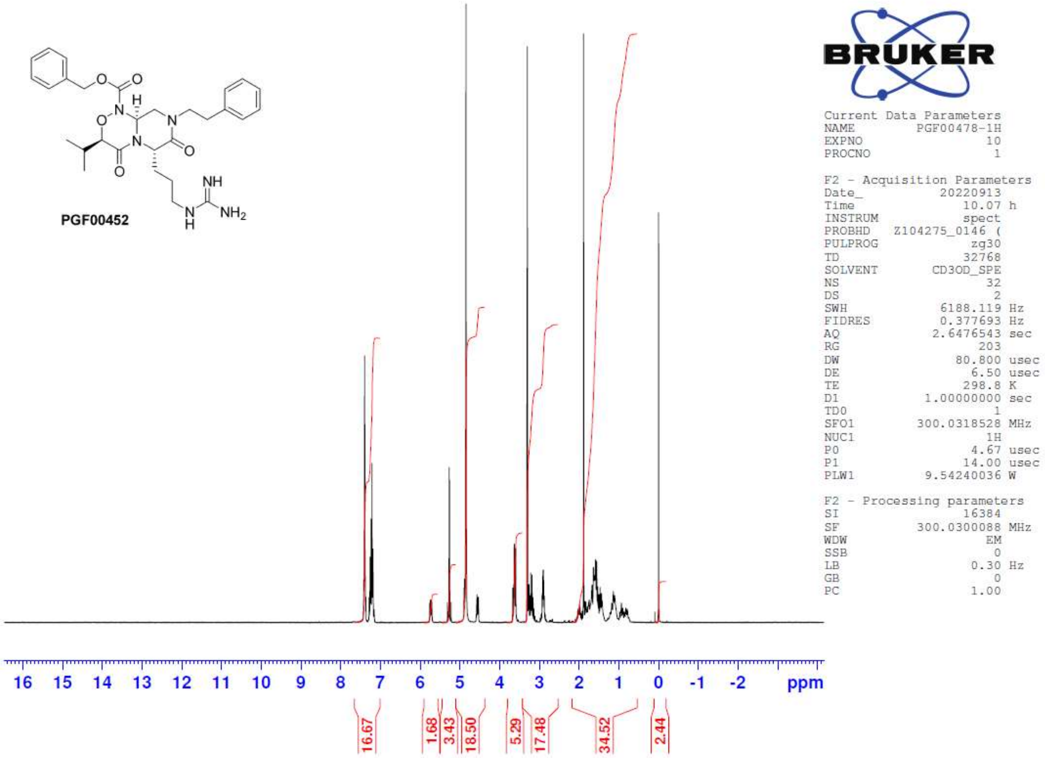

Supplementary Figure S7. ^{1}H NMR spectrum of **PGF00452**.

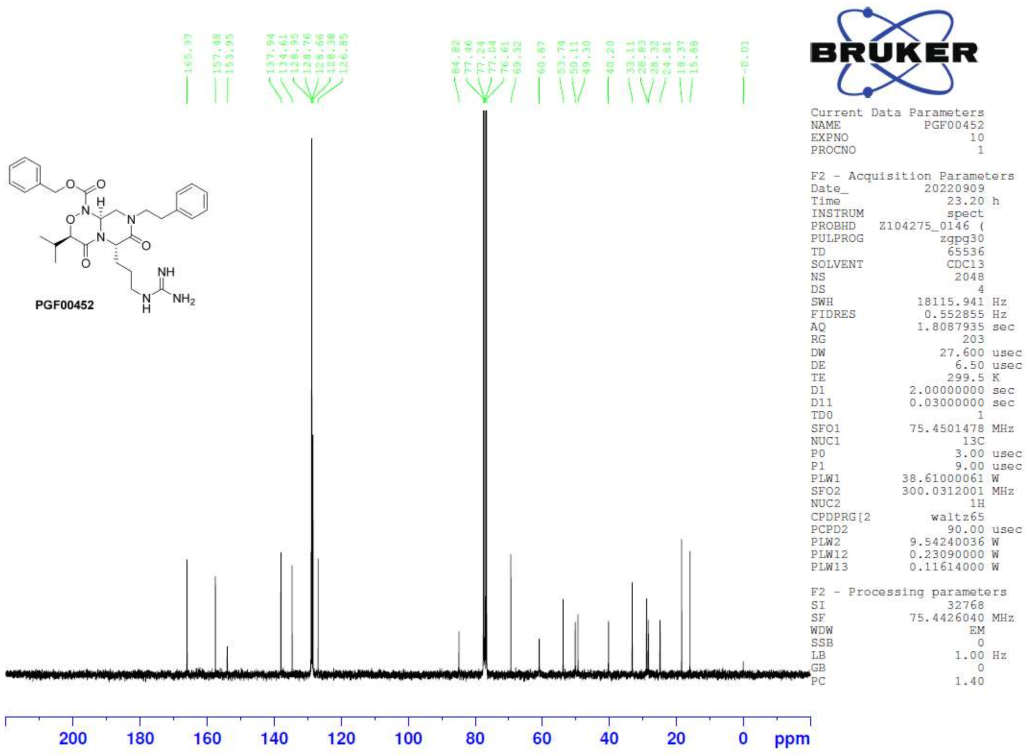

Supplementary Figure S8. ^{13}C NMR spectrum of **PGF00452**.

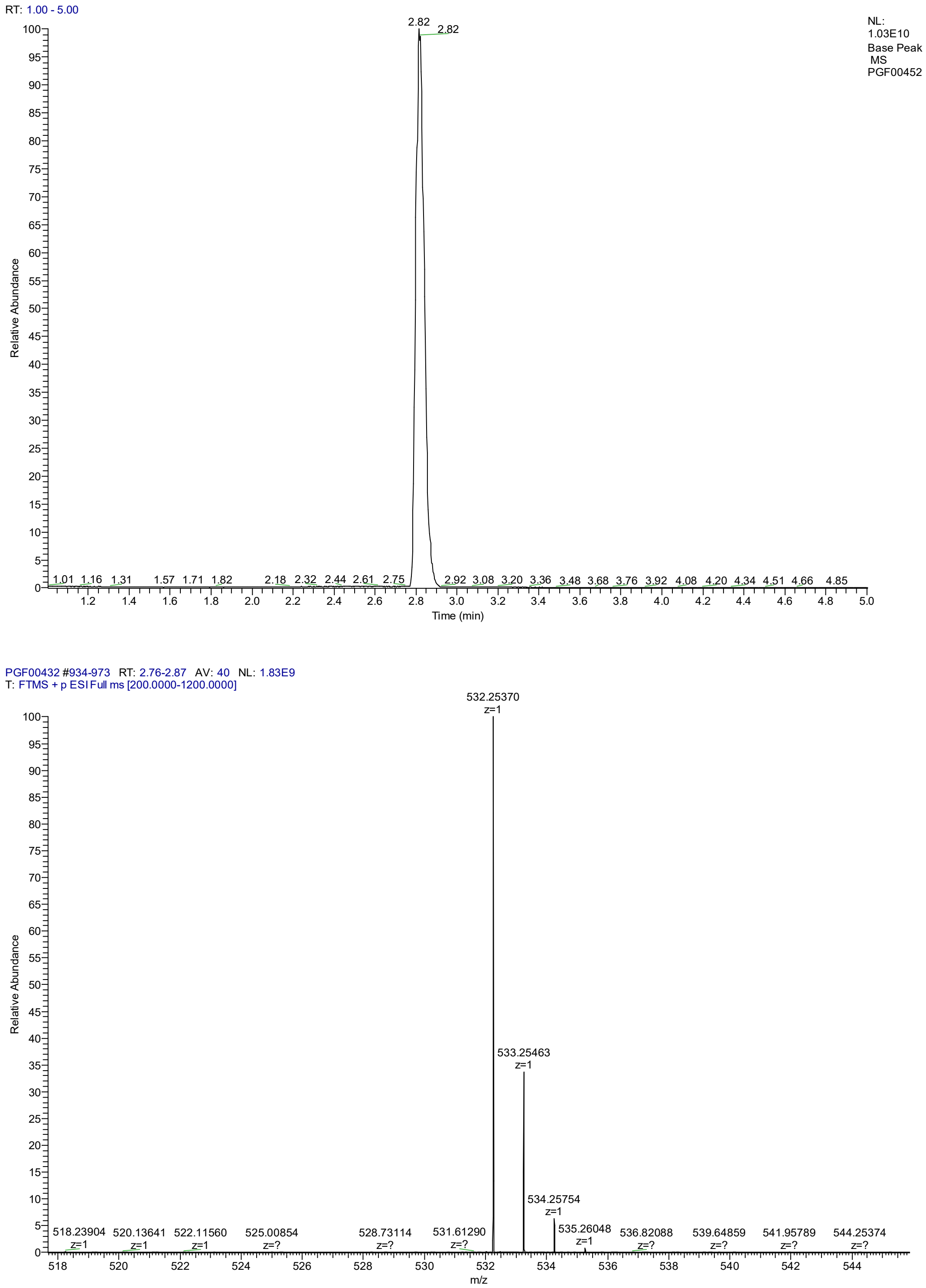

Supplementary Figure S9. HRMS spectrum of **PGF00452**.

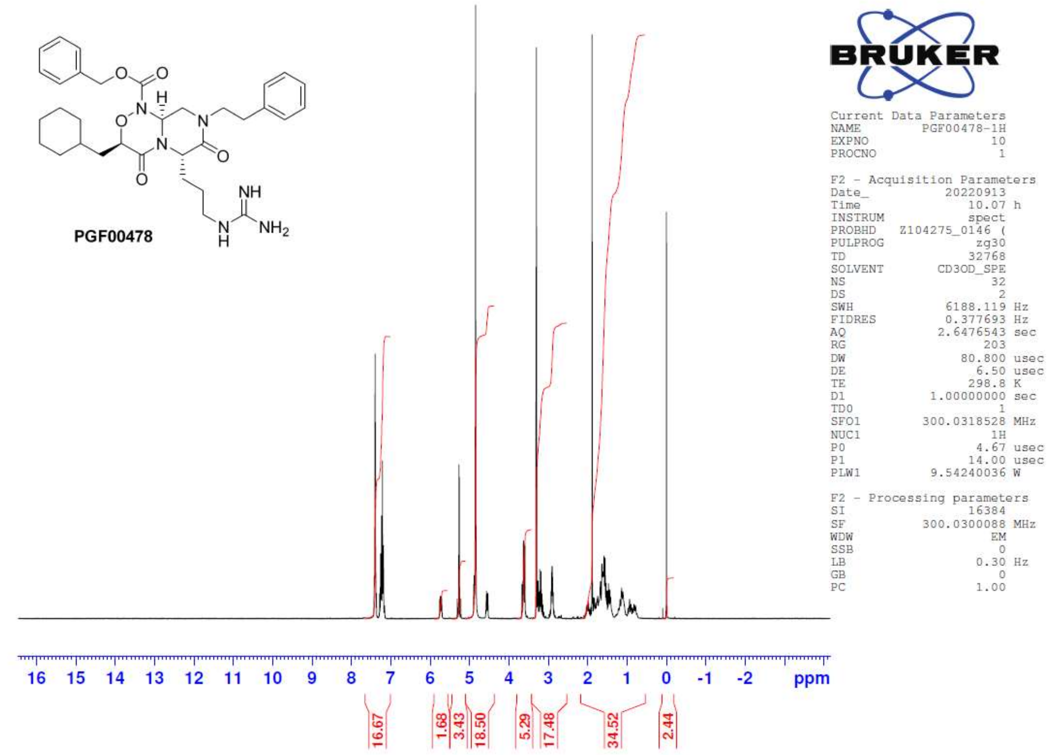

Supplementary Figure S10. ^{1}H NMR spectrum of **PGF00478**.

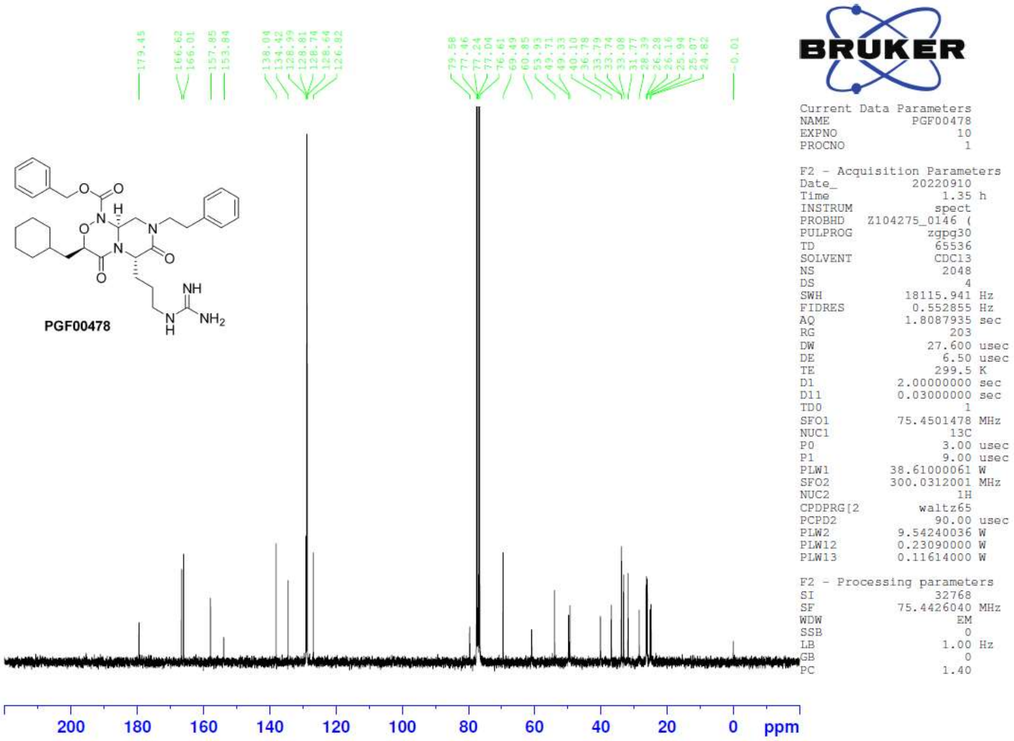

Supplementary Figure S11. ^{13}C NMR spectrum of **PGF00478**.

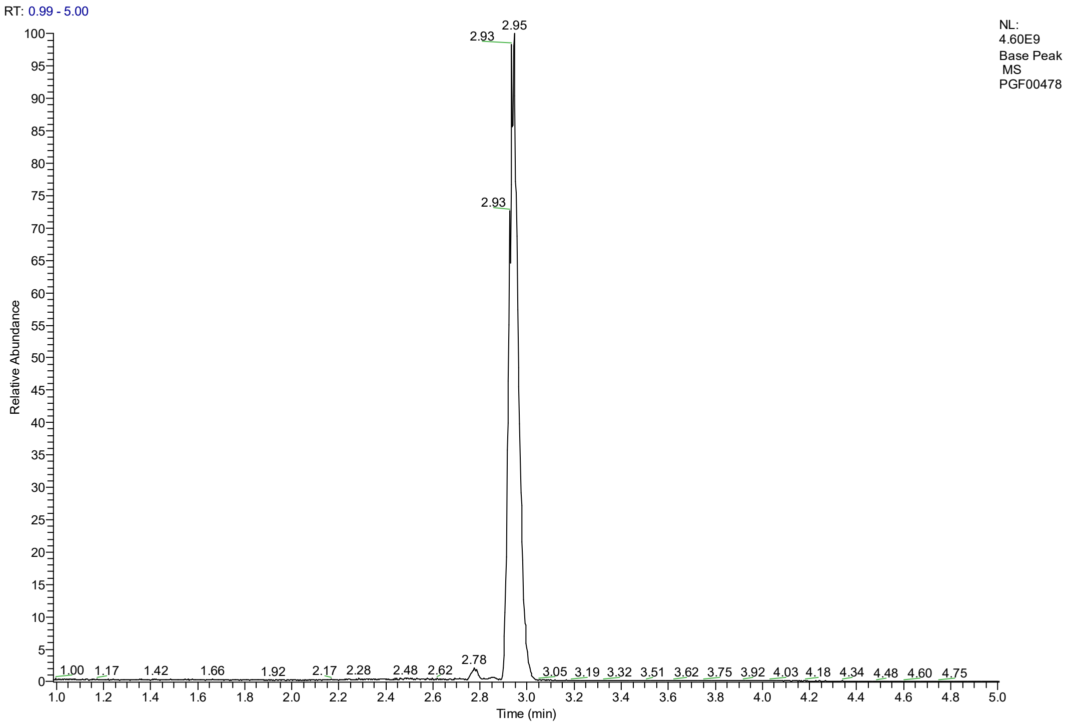

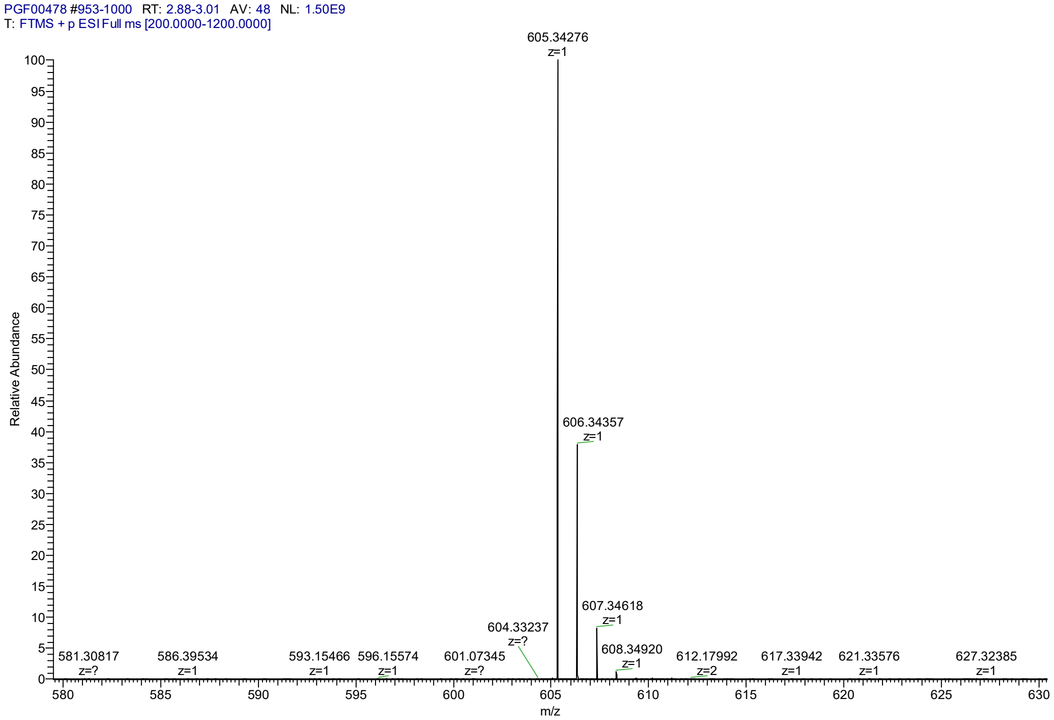

Supplementary Figure S12. HRMS spectrum of **PGF00478**.

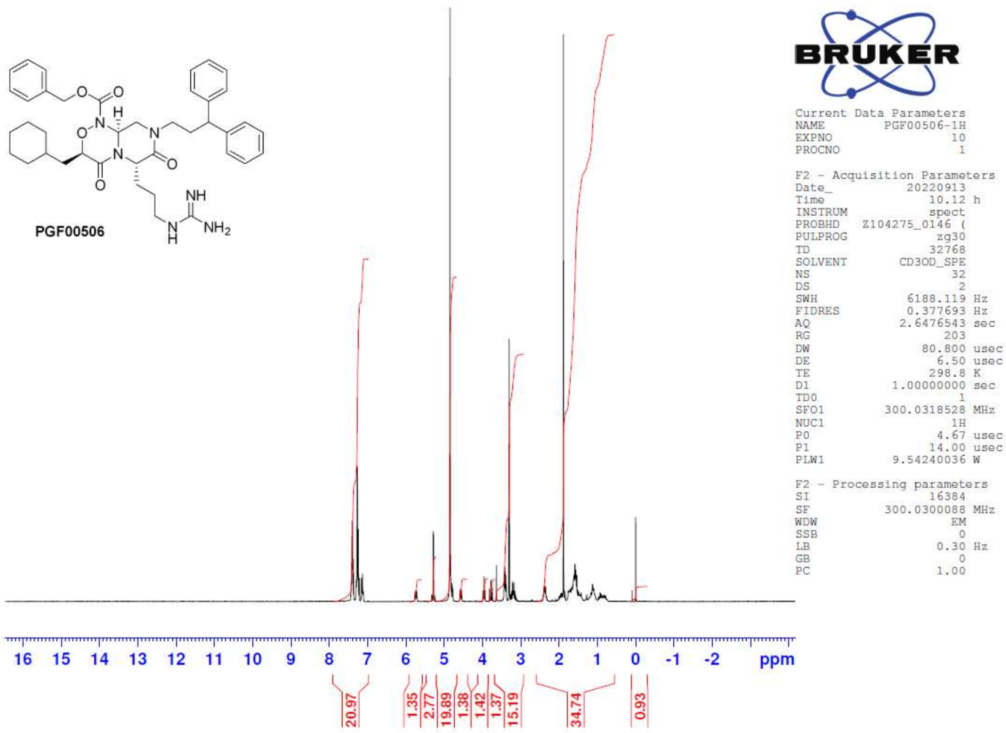

Supplementary Figure S13. ^{1}H NMR spectrum of **PGF00506**.

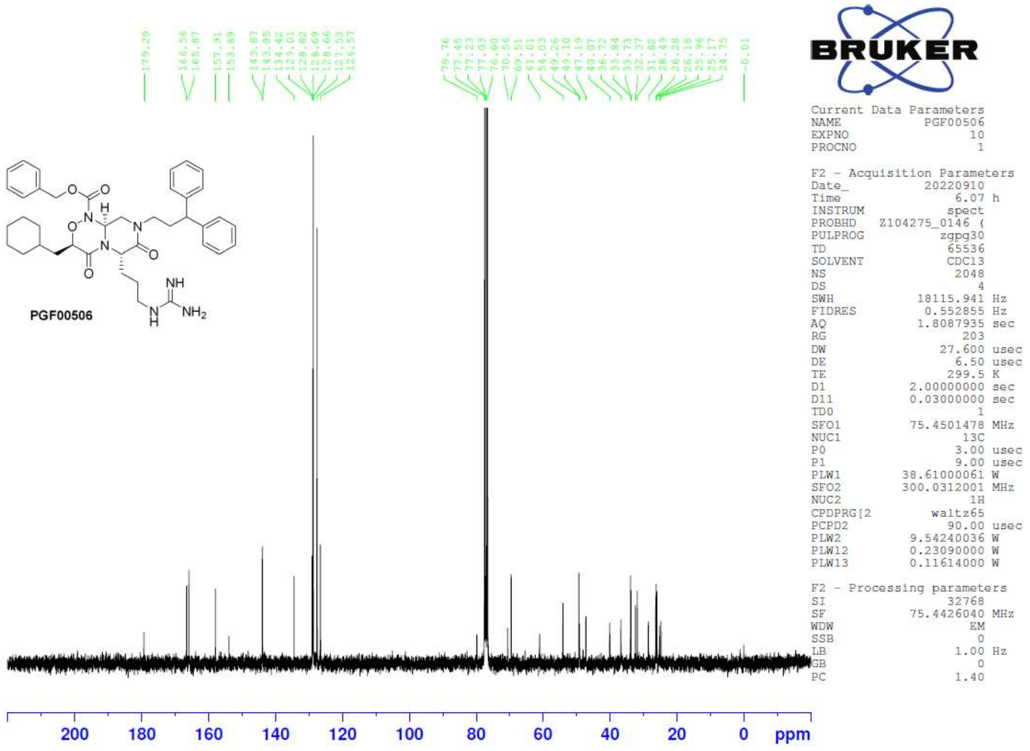

Supplementary Figure S14. ^{13}C NMR spectrum of **PGF00506**.

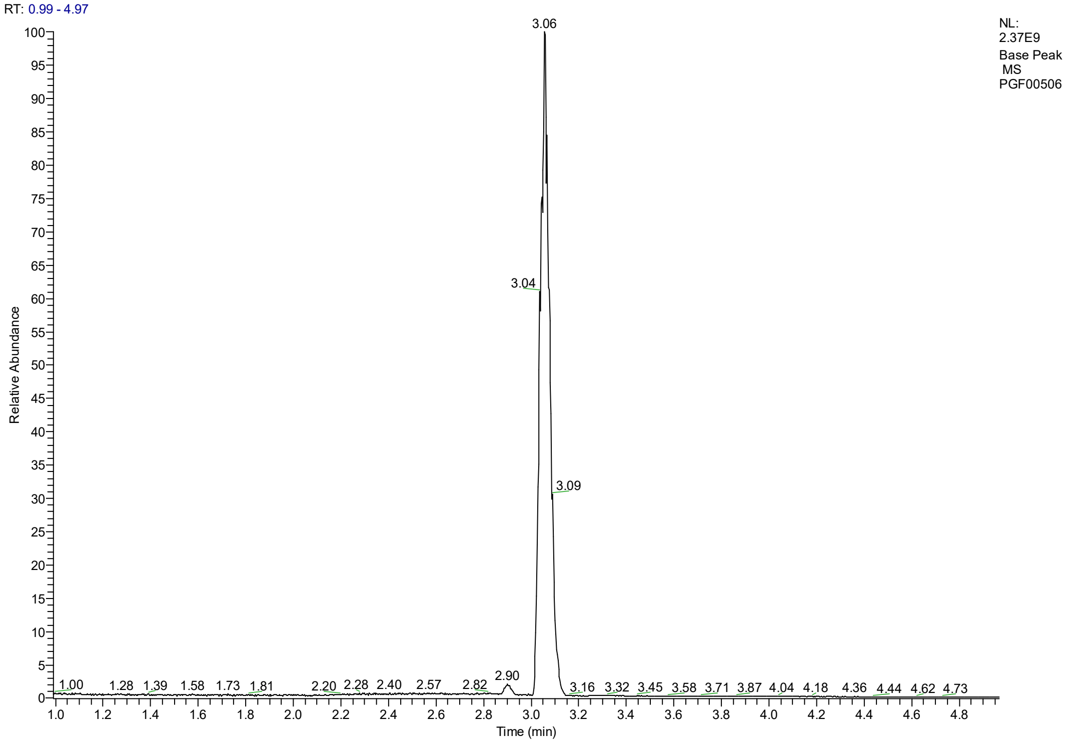

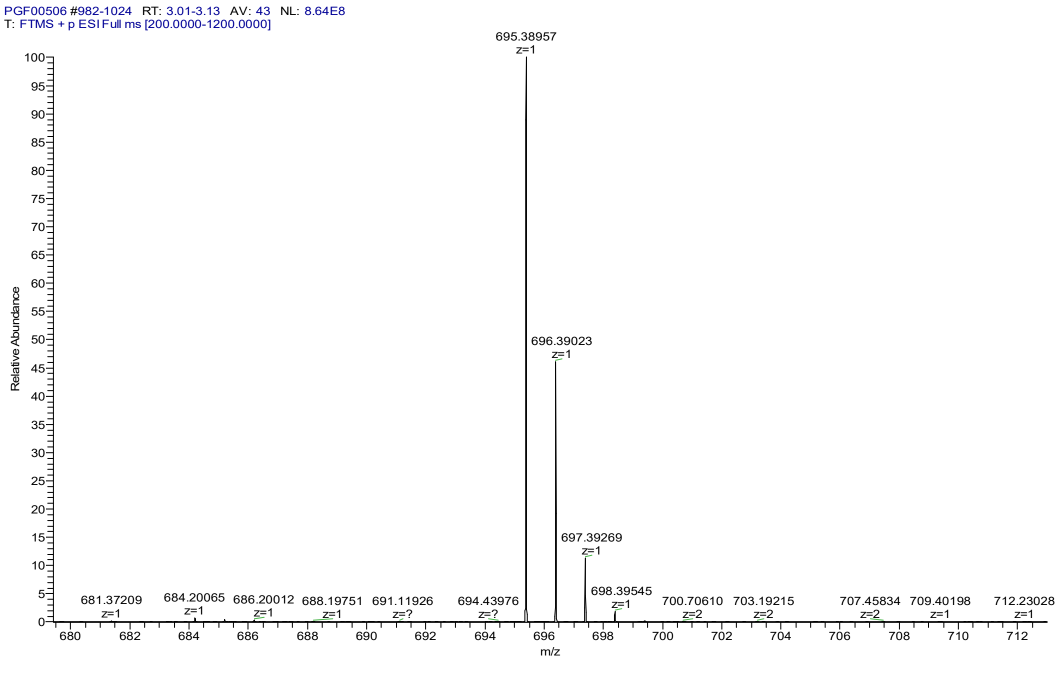

Supplementary Figure S15. HRMS spectrum of **PGF00506**.
